# Quinazoline-2,4(1H,3H)-dione derivatives as new class of CB1 Agonists: A pharmacophore-based virtual screening workflow and drug discovery

**DOI:** 10.12688/f1000research.171433.3

**Published:** 2026-03-11

**Authors:** Abdellah EL AISSOUQ, MOURAD STITOU, Mohamed Enneiymy, Said El Rhabori, Hicham Zaitan, Abdelkrim Ouammou, Fouad Khalil

**Affiliations:** 1 Universite Sidi Mohamed Ben Abdellah Faculte des Sciences et Techniques de Fes, Fes, Fes-Boulemane, Morocco; 2 Universite Ibn Zohr Faculte des Sciences Agadir, Agadir, Souss-Massa-Draa, Morocco; 3 Universite Sidi Mohamed Ben Abdellah Faculte des Sciences Dhar El Mahraz-Fes, Fes, Fes-Boulemane, Morocco

**Keywords:** CB1 agonists, pharmacophore based virtual screening, lead discovery, quinazoline-2, 4(1H, 3H)-dione derivatives

## Abstract

**Background:**

The cannabinoid 1 (CB1) receptor is the primary target of Δ ^9^-tetrahydrocannabinol (Δ ^9^-THC), the psychoactive component of cannabis sativa (commonly known as “kif” in Morocco).

**Methods:**

Here, we identified novel CB1 agonists using virtual screening approaches. First, we developed a pharmacophore model based on the known CB1 agonist AM11542 and screened a database of over three million compounds. Molecular docking using AutoDock Vina identified 61 hits with binding affinities of less than -9.00 Kcal/mol. Subsequent ADME-Tox (absorption, distribution, metabolism, excretion, and toxicity) analysis narrowed the selection to 18 promising candidates.

**Results:**

Among these, three agonists exhibited strong characteristics, including a favorable inhibition constant (Ki) and key hydrogen-bond interactions with critical residues in the CB1 binding pocket: PUBChem157251136 (Ki=2.09 nM), ZINC64438485 (Ki= 0.262 nM) and ZINC64438506 (Ki =0.244 nM). These agonists formed stable hydrogen bonds with CB1 binding pocket residues (Ser383, Ser173, His178 and Thr197). Molecular dynamics simulations (100 ns, GROMACS) demonstrated structural stability (RMSD < 1 nm) and low conformational flexibility (RMSF < 1 nm) for all complexes. MM-GBSA binding free energy calculations further confirmed the thermodynamic stability of all complexes, with interaction energies ranging from -30.59 to -49.98 kcal/mol. These comprehensive simulations confirm that all identified agonist complexes maintain stable binding conformations with optimal interaction profiles characteristic of CB1 receptor activation.

**Conclusion:**

These results could pave the way for researching and developing new quinazolinz-2, 4(1H, 3H)-Dione derivatives as a new class of CB1 receptor agonists.

## I. Introduction

Cannabinoid receptors are classified as G protein-coupled receptors which belong to a family known as the endocannabinoid system, a master regulator of numerous physiological pathways throughout the human body. ^1^ There are two categories of cannabinoid receptors, known as CB1 and CB2. The CB1 receptor is found throughout multiple organs of the body; it is present in the digestive tract, liver, pancreas, and musculature, in addition to its primary site of localization being in the brain. ^2^ In fact, CB1 is the most highly expressed receptor in the brain relative to other receptors examined, possessing 7 transmembrane domains with G protein coupling. The CB1 receptor mediates cannabinoid-induced psychotropic effects. The CB2 receptor is more so located within immune cells, where it exerts its immunomodulatory role.

Cannabinoids are a class of chemical compounds which are championed globally for their psychoactive and physiologic whole body functions; for at least 5,000 years, ^3, 4^ mankind has been able to capitalize on the value of cannabinoids. One such natural source of cannabinoids is cannabis, known as “kif” in Moroccan culture, a natural product with a plethora of phytoconstituents including Δ9-tetrahydrocannabinol (THC). The phytochemical THC exerts its psychotropic effects by acting at the CB1 receptor, which also serves as the primary receptor for the endogenous endocannabinoids anandamide (AEA) and 2 arachidonoylglycerol (2-AG). ^5^ Activation of the CB1 receptor upregulates potassium channel currents and calcium ion channel polarization, making receptor signaling dose dependent and responsive to treatment with pertussis toxin. ^6^ In addition, CB1 can exist as a homodimer and/or form complexes as heterodimers or heterooligomers with other G Protein-Coupled Receptors (GPCRs). Lastly, the CB1 receptor is in a complex with GABAergic and glutamatergic cells, and therefore, CB1 receptor stimulation decreases release from GABAergic and glutamatergic cells. ^7^

**Figure 1.**
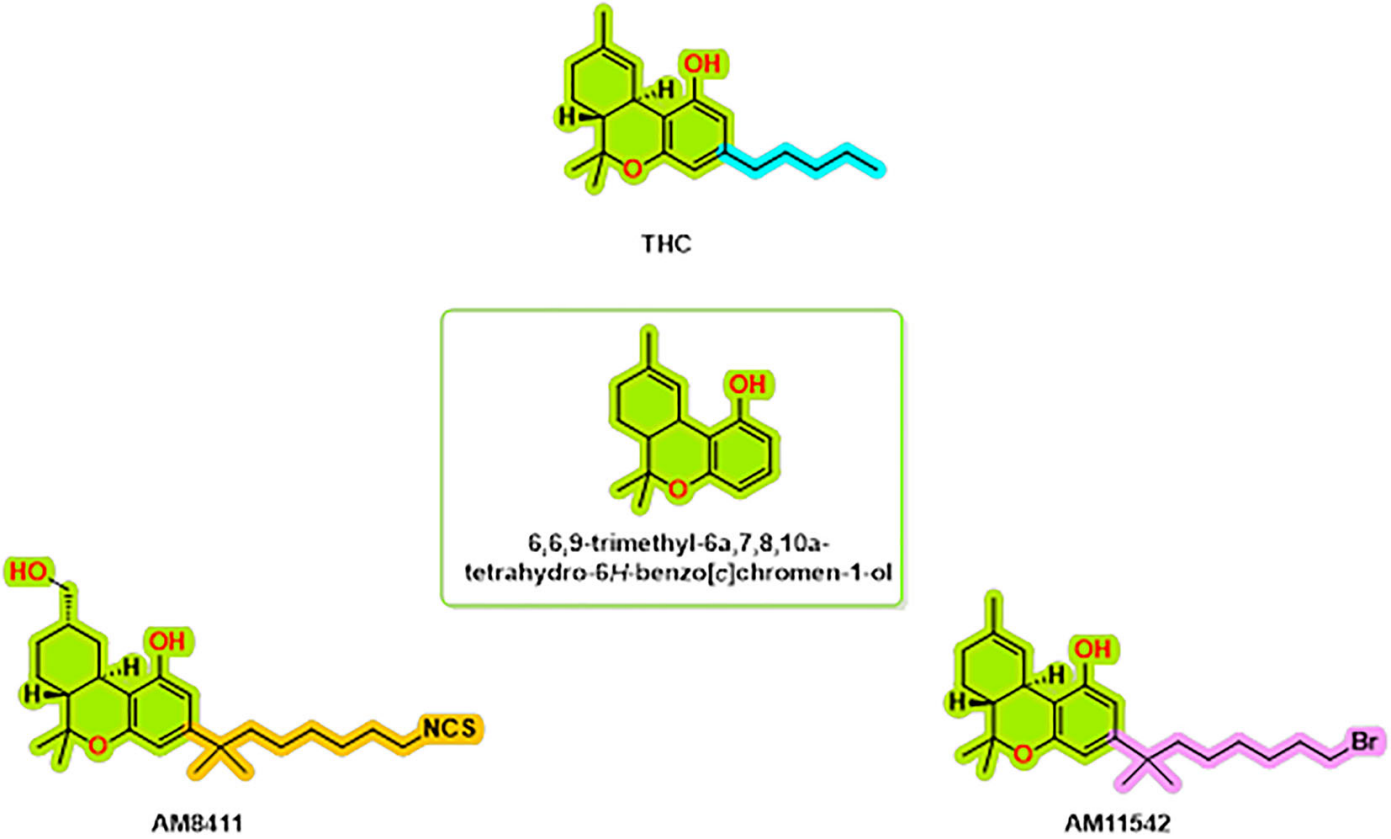
CB1 receptor agonist chemical structures.

The structural complexes of the cannabinoid receptor CB1 with THC analogs are an important topic of research to help us understand the molecular interactions that underlie cannabinoid signaling. Thus far, the repertoire of known complexes of the CB1 receptor includes the structure of CB1, bound to AM8411, ^8^ CP55940 ^9^ and AM11542 ^10^ ( Figure 1). These structures have a resolution of 2.8-3.4 Å and provide a highly informative account of the binding modes and associated conformational changes that can accompany receptor activation. Several important interacting residues have been found to be conserved between agonist-bound structures, including hydrophobic interactions with LEU193 ^3.29^, VAL196 ^3.32^, PHE200 ^3.36^, TYR275 ^5.39^, LEU276 ^5.40^, TRP279 ^5.43^, TRP356 ^6.48^, LEU359 ^6.51^ and MET363 ^6.55^. ^11^ These observations further emphasize the importance of these residues in their role of stabilizing binding of agonists to the receptor, while also promoting receptor activation.

There were also three structures solved by means of cryo-electron microscopy (cryo-EM) that included full active states along with the Gi1-2 G protein subunit. ^12^ The AM11542-CB1 complex is a crystalline structure and had a fusion protein, flavodoxin, which contributes to stabilization of TM6 in an active confirmation. ^11^ All of the structural coordinates cover the full CB1 protein sequence, covering anywhere between approximately 58% and 62% of each residues in all active structures. ^8^ The fact that the coordinates do not cover the full sequence is most likely due to the flexibility imparted by both the N and C-terminus and the long three intracellular loops (ICL3). These structural descriptions not only allow constructs for determining the molecular mechanisms for THC analog activation of the CB1 receptor but also create opportunities for future studies.

In recent years the harmonization of approaches have created several avenues to create CB1 receptor agonists with less adverse effects. This study will previously develop a CB1 agonist pharmacophore model that was used as a virtual screening tool for unique/novel class of CB1 agonists. Using the pharmacophore this was able to screen over 300 million hits, of compounds, parsed from 11 different databases and were able to identify many candidates that have not been screen for cannabinoid receptor binding assay. This highlights the usefulness of computational strategies to find novel therapeutics that have the potential to provide safer alternatives.

## II. Materials and method

### 1. Pharmacophore modeling and virtual screening

**Figure 2.**
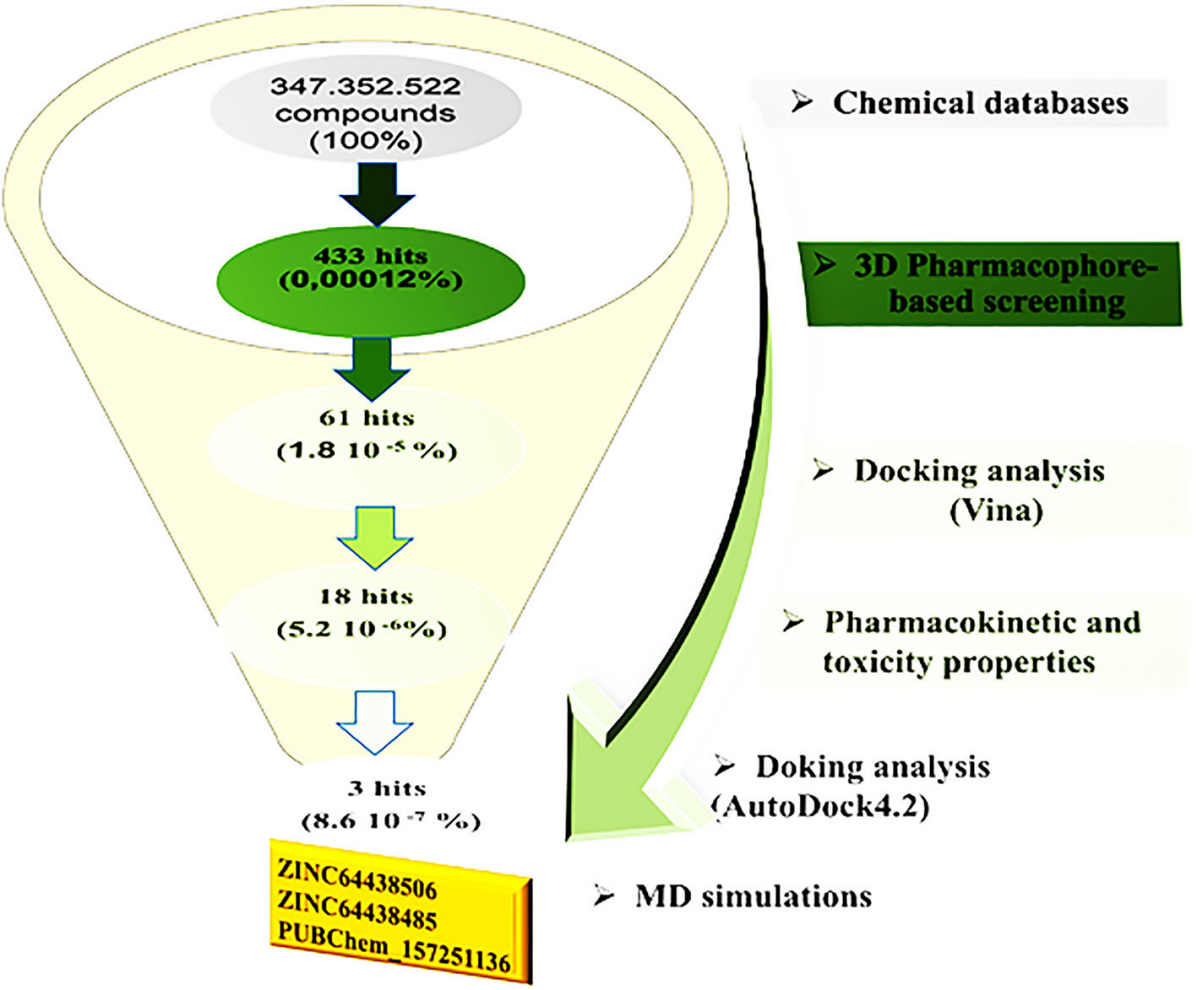
General workflow used in the present study for identifying new CB1 agonists.

Pharmacophore based virtual screening were performed using the Pharmit web interface ( http://pharmit.csb.pitt.edu/), which has a number of built-in databases that provide access to comprehensive data; Molprot (4,742,020 molecules), ChEMBL34 (2,264,112 molecules), ZINC (13,127,550 molecules), ChemDiv (1,456,120 molecules), ChemSpace (50,181,678 molecules), Enamine (4,117,328 molecules), MCULE (39,843,637 molecule), MCULE-ULTIMATE (126,471,502 molecules), NCI Open Chemical Repository (52,237 molecules), LabNetwork (1,794,286 molecules), and PubChem (103,302,052 molecules). The pharmacophore model was constructed using selected PDB code 5XRA from the RCSB Protein Data Bank ( http://www.rcsb.org/structure/5xra), with agonist AM11542. The model utilized a pharmacophore framework built on five features, adhering to the default parameters of the Pharmit server. The Pharmit filters were applied based on the Lipinski rule of five and Veber’s rule to refine the screening process and identify the most selective CB1 agonist. Figure 2 illustrates the multi-step virtual screening process used in this work.

### 2. ADMET profiling

The ADMET (Absorption, Distribution, Metabolism, Excretion, and Toxicity) filter is one of the phases of the drug discovery and development process. ^13– 15^ This allows researchers to determine potentially druggable drug-like properties and toxicology early on to assess which compounds will be effective drugs with safety potential. Thus, relative to virtual screening, such a filter can detect adverse properties that would fail at much later stages, such as insufficient absorption, excessive toxicity, and/or suboptimal bioavailability. Two of the trusted websites relied upon to evaluate such properties based upon lipophilicity, logP, solubility, etc., are SwissADME ( http://www.swissadme.ch) and pKCSM ( https://biosig.lab.uq.edu.au/pkcsm/prediction). Therefore, when these are filtered out early on, time and costs will be effectively saved down the line.

### 3. Molecular docking studies

To estimate the binding affinities of the 433 highest-ranked compounds to CB1, molecular docking was carried out using AutoDock Vina integrated within the PyRx platform. The crystal structure of CB1 (PDB ID: 5XRA), with a resolution of 2.8 Å, ^8, 16, 17^ was retrieved from the Protein Data Bank. Before docking, water molecules and the native ligand were removed from the protein, followed by the addition of polar hydrogen atoms and Kollman charges. The prepared protein structure was then energy-minimized using UCSF Chimera and saved in PDBQT format.

Prior to docking, all 433 compounds underwent pre-optimization utilizing the Universal Force Field (UFF) in combination with the conjugate gradient algorithm. ^18^ Optimization parameters were set to 2000 total steps, with updates occurring every step, and the process was programmed to terminate once the energy difference fell below 0.01 kcal/mol. ^19^ After optimization, the compounds were converted to PDBQT format and docked at specific binding site coordinates (x = -42.052, y = -164.338, z = 306.631). Molecules demonstrating the lowest binding energies and root-mean-square deviation (RMSD) values below 2 Å were selected for subsequent analyses.

### 4. Molecular dynamics simulations

Molecular dynamics (MD) simulations were carried out on the top-ranked docking conformations using the GROMACS 2024.4 software package, employing the CHARMM27 all-atom force field. ^20^ The topology files for the CB1 receptor were generated using the pdb2gmx tool, ^21^ while the ligand topologies were prepared through the CHARMM General Force Field (CGenFF) using the Param-Chem server. ^22– 24^ Each receptor-ligand complex was embedded in a triclinic simulation box and solvated with TIP3P water molecules, maintaining a minimum distance of 1.0 nm from the box boundaries. To neutralize the systems, Na ^+^ and Cl ^-^ ions were added accordingly.

Prior to the production runs, energy minimization was performed using the steepest descent algorithm for 50,000 steps to eliminate steric clashes and ensure system stability. Subsequently, two equilibration phases were conducted: first under the NVT ensemble to stabilize temperature, followed by the NPT ensemble to equilibrate pressure and density. Input files for both equilibration and production stages were customized to adjust parameters such as trajectory-saving intervals, energy monitoring, and other essential simulation settings. Finally, each ligand-receptor system underwent 100 ns of production MD simulation at a constant temperature of 300 K, pressure of 1 bar, and a time step of 2 femtoseconds.

### 5. MM/GBSA method

The binding free energy was calculated via MM/GBSA (Molecular Mechanics Generalized Born Surface Area). ^25^ This approach utilizes force fields of molecular mechanics and an implicit solvent approach to conclude stability and binding tendencies of molecular complexes. It is particularly useful for classifying ligands and understanding overall energetic contributions of molecular recognition. The binding free energy ΔGbind of a complex can be calculated as follows:

$${\mathrm{\Delta G}}_{\text{bind}}={\mathrm{\Delta E}}_{\mathrm{MM}}+{\mathrm{\Delta G}}_{\text{solv}}–\mathrm{T\Delta S}$$ (Eq.1)

Where:

$${\mathrm{\Delta E}}_{\mathrm{MM}}=E_{\mathrm{MM}}^{\text{complex}}-\left(E_{\mathrm{MM}}^{\text{receptor}}+E_{\mathrm{MM}}^{\text{ligand}}\right)$$ (Eq.2)

$${\mathrm{\Delta G}}_{\text{solv}}=G_{\text{solv}}^{\text{complex}}-\left(G_{\text{solv}}^{\text{receptor}}+G_{\text{solv}}^{\text{ligand}}\right)$$ (Eq.3)

$$G_{\text{solv}}=G_{\mathrm{GB}}+G_{\mathrm{SA}}$$

Where TΔS, ΔE _MM_ and ΔG _sol_ are the conformational entropy upon binding, the changes of the gas phase molecular mechanism (MM) energy and the solvation free energy, respectively.

## III. Results and discussion

### 1. Pharmacophore

**Figure 3.**
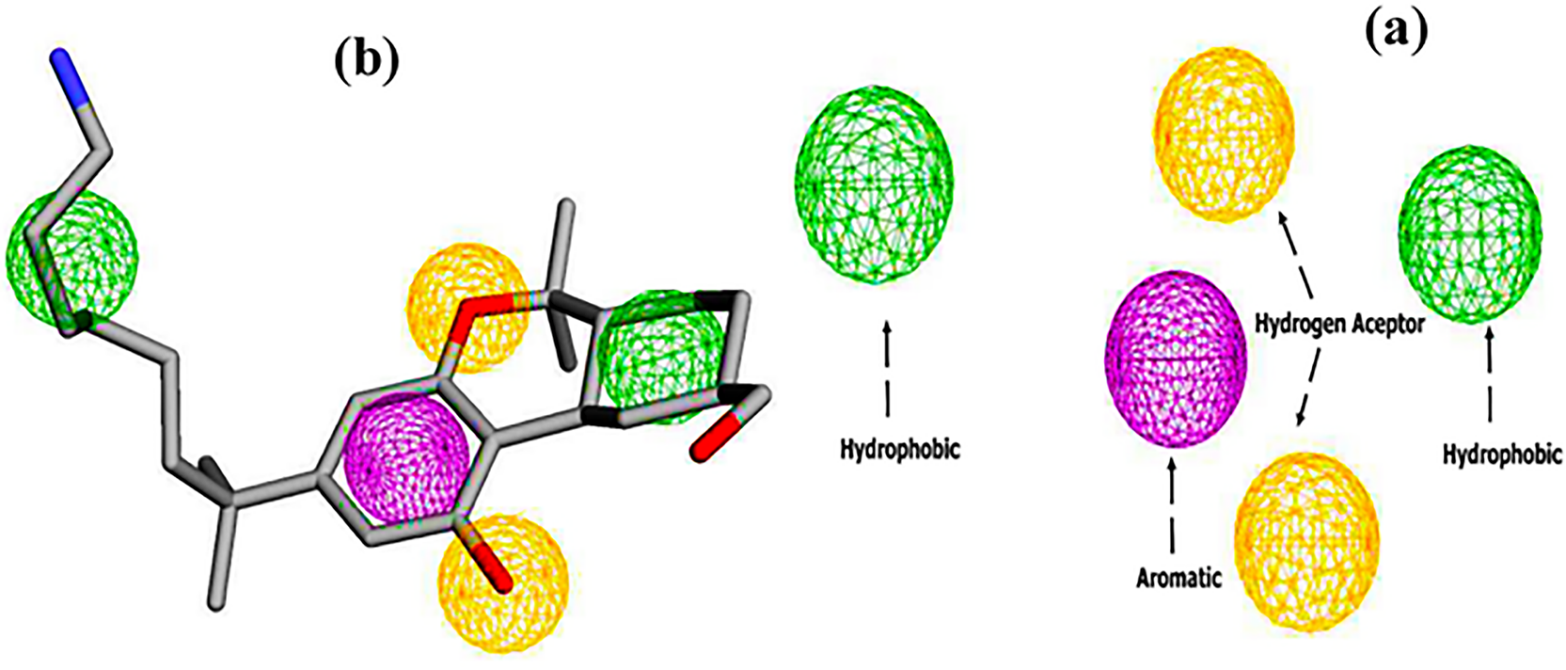
Pharmacophore features (a) two hydrogen bond acceptors (2HBA, yellow color), two hydrophobic groups (2HY, Green color), and one aromatic group (1AR, purple color), (b) Pharmacophore feature mapping of AM11542 agonist.

The pharmacophore model of the CB1 receptor agonist was generated based on the AM11542 agonist. The results are shown in Figure 3. A five-point model with 1 aromatic, 2 hydrogen bond acceptors and 2 hydrophobic regions was generated using the Pharmit web server. ^26^

Subsequently, virtual screening was performed across 11 databases, identifying 433 compounds containing molecular groups that matched the pharmacophore mode.

### 2. Virtual screening

Pharmacophore-based virtual screening was performed using information from the previous pharmacophore model. Approximately 300 million compounds from the Pharmit database were filtered. The criteria for filtering the library were as follows: molecular weight limits were set at 250-500 Da, the maximum number of hydrogen bond donors was less than 4, the maximum number of hydrogen bond acceptors was less than 9, the maximum number of rotatable bonds was less than 9, Log P was between 2 and 5, and polar surface area was less than 140 Å. The results are computed and classified according to different criteria such as energy minimization. The 433 top-ranked compounds are presented in Table 1.

**Table 1.** Pharmacophore-based virtual screening of compounds from 11 databases on the Pharmit server.

Pharmit database	Molecules	Hits
Molprot	4,742,020	**58**
ChEMBL34	2,264,112	**134**
ZINC	13,127,550	**32**
ChemDiv	1,456,120	**76**
ChemSpace	50,181,678	**0**
Enamine	4,117,328	**5**
MCULE	39,843,637	**23**
MCULE-ULTIMATE	126,471,502	**0**
NCI Open Chemical Repository	52,237	**0**
LabNetwork	1,794,286	**63**
PubChem	103,302,052	**42**
**Total**	**347.352.522**	**433**

Next, molecular docking analysis was performed using AutoDock Vina, implemented in the PyRx software. Compounds with a binding affinity of less than - 9.00 kcal/mol were selected for further analysis, while the others were excluded. Additionally, the predicted binding modes were required to have a root-mean-square deviation (RMSD) of less than 2Å when superimposed onto the native agonist (AM11542). The top 61 selected compounds are listed in Table 2, while the remaining candidates are provided in **Table S1** (see supplementary data).

**Table 2.** Top 61 compounds selected based on docking binding affinity (binding affinity < -9.00 Kcal/mol) and rmsd (rmsd < 2 Å) values.

Code	Binding affinity (kcal/mol)	rmsd	Code	Binding affinity (kcal/mol)	rmsd
ZINC35377792	-10.526	1.766	ZINC21525754	-9.349	1.086
ZINC17286185	-10.487	1.825	ZINC35377780	-9.311	1.265
ZINC45898833	-10.415	1.964	PubChem-16352732	-9.308	0.823
ZINC35562518	-10.410	1.055	PubChem-136659176	-9.294	1.082
ZINC35377809	-10.202	1.239	PubChem-135405892	-9.292	1.457
ZINC64438506	-10.189	1.395	PubChem-126853168	-9.271	1.275
PubChem-156469643	-10.074	1.867	PubChem-25220434	-9.254	1.924
PubChem-89734004	-10.054	1.291	ZINC20138980	-9.240	1.274
PubChem-50762870	-10.049	1.717	ZINC169	-9.236	1.227
PubChem-86747888	-10.035	1.776	ZINC96385691	-9.222	1.955
ZINC45899482	-10.006	1.796	ZINC65196494	-9.217	0.750
ZINC64438485	-9.944	1.964	MolPort-009-386-250	-9.208	1.702
PubChem-121231416	-9.858	1.197	PubChem-41119771	-9.192	1.542
ZINC21797190	-9.850	1.617	ZINC20113894	-9.173	0.794
ZINC45899726	-9.828	1.718	ZINC20113894	-9.173	0.794
ZINC45900106	-9.824	1.499	ZINC35377767	-9.130	1.670
PubChem-53794837	-9.820	1.352	PubChem-71600230	-9.127	1.653
ZINC45899547	-9.785	1.651	ZINC45899386	-9.127	1.416
CHEMBL1652254	-9.763	1.547	ZINC35377767	-9.121	1.351
ZINC2245716	-9.652	1.160	ZINC13365292	-9.100	1.219
ZINC45900103	-9.615	1.977	LN00379431	-9.098	1.083
ZINC35377763	-9.518	1.176	PubChem-3750748	-9.095	1.627
PUBChem 157251136	-9.492	1.362	ZINC4034881	-9.089	1.300
ZINC09598984	-9.489	1.654	ZINC65196500	-9.068	1.934
ZINC35377731	-9.485	1.417	ZINC100771598	-9.068	1.445
ZINC 35377767	-9.465	1.474	PubChem-25352696	-9.067	1.172
ZINC 21723065	-9.463	1.471	PubChem-91428044	-9.045	1.614
PubChem-42810820	-9.454	1.742	ZINC35377731	-9.034	1.382
ChemDiv-C260-2692	-9.424	1.461	ZINC33057775	-9.027	1.058
PubChem-135869534	-9.413	1.985	ZINC96385592	-9.010	1.069
PubChem-91487881	-9.353	1.714			

### 3. Toxicity filters

The ranked compounds from docking analysis were evaluated for potential toxicity, including an AMES toxicity test, an acute oral toxicity test in rats (LD _50_), a skin sensitization test and a maximum tolerated dose analysis. Highly toxic compounds are not considered in further studies. The top selected compounds are presented in Table 3. The other are presented in the supplementary data ( **Table S2).**

**Table 3.** *In silico* prediction of Skin Sensitisation. AMES toxicity, oral rat acute toxicity (LD _50_), and Max Tolerated dose in humans.

Code	Skin Sensitisation	AMES toxicity	Oral Rat Acute Toxicity (LD _50_ (mol/kg))	Max. tolerated dose (human) (Log mg/kg/day)
ZINC35377792	No	No	2.785	0.412
ZINC17286185	No	No	2.474	0.222
ZINC45898833	No	No	2.617	0.328
ZINC35562518	No	No	2.441	0.215
ZINC64438506	No	No	2.744	0.685
PubChem-156469643	No	No	2.904	-1.12
PubChem-89734004	No	No	3.02	-0.825
PubChem-86747888	No	No	3.001	-0.913
ZINC64438485	No	No	2.816	0.381
PubChem-121231416	No	No	2.818	-0.595
ZINC21797190	No	No	3.085	0.61
ZINC45899726	No	No	2.748	0.647
PubChem-53794837	No	No	2.569	-0.725
CHEMBL1652254	No	No	3.004	0.428
ZINC2245716	No	No	2.469	0.586
PUBChem 157251136	No	No	2.812	-0.095
Zinc35377731	No	No	2.863	0.328
Zinc21723065	No	No	2.544	0.463
PubChem-42810820	No	No	2.73	0.562
ChemDiv-C260-2692	No	No	2.475	0.6
PubChem-135869534	No	No	3.077	0.604
PubChem-91487881	No	No	2.433	-0.088
ZINC21525754	No	No	2.753	-0.134
PubChem-16352732	No	No	2.218	0.971
PubChem-136659176	No	No	2.242	0.572
PubChem-135405892	No	No	3.075	0.607
PubChem-126853168	No	No	2.67	0.443
ZINC65196494	No	No	3.354	-0.164
PubChem-41119771	No	No	2.38	0.016
ZINC20113894	No	No	2.574	0.429
ZINC20113894	No	No	2.574	0.429
PubChem-71600230	No	No	2.048	0.395
ZINC13365292	No	No	2.604	0.134
LN00379431	No	No	2.567	0.663
PubChem-3750748	No	No	3.038	-0.137
ZINC4034881	No	No	2.576	0.549
ZINC65196500	No	No	3.156	-0.141
ZINC100771598	No	No	3.008	0.471
PubChem-25352696	No	No	2.552	1.059
PubChem-91428044	No	No	1.439	0.563
ZINC35377731	No	No	2.863	0.328
ZINC33057775	No	No	2.503	0.769

As described in Table 3, all selected compounds exhibited no Skin Sensitisation and no AMES toxicity. Additionally, the LD _50_ values for oral rat toxicity, ranging from 1.439 to 3.354 mol/kg, suggest moderate acute toxicity levels, consistent with safety margins suitable for therapeutic use. Moreover, the maximum tolerated dose in humans’ ranges from -1.12 to 1.059 Log mg/kg/day.

### 4. Physicochemical properties and bioavailability

Physicochemical properties were evaluated using Lipinski’s rule of five, ^27^ Ghose’s rule, ^28^ Veber’s rule, ^29^ Egan’s rule ^30^ and Muegge’s rule, ^31^ with results detailed in Table 4. Based on these guidelines, it is suggested that for a compound to be effectively absorbed and administered orally, it must meet specific physicochemical parameters. These criteria serve as essential benchmarks for assessing the compound’s potential for bioavailability and oral absorption. Compounds with two or more violations are not considered in the further analysis (see **Table S3** in supplementary data).

**Table 4.** Physico-chemical properties based on the rules of Lipinski, Ghose, Veber, Egan and Muegge for the highest ranked compounds.

Code	Lipinski #violations	Ghose #violations	Veber #violations	Egan #violations	Muegge #violations
ZINC17286185	0	1	0	0	0
ZINC35562518	0	1	0	0	0
ZINC64438506	0	1	0	0	0
PubChem-156469643	0	0	0	0	1
PubChem-89734004	0	0	0	0	0
PubChem-86747888	0	0	0	0	1
ZINC64438485	0	1	0	0	0
PubChem-121231416	0	0	0	0	0
ZINC21797190	0	0	0	0	0
PubChem-53794837	0	0	0	0	1
CHEMBL1652254	0	0	0	0	1
PUBChem 157251136	0	0	0	0	0
PubChem-42810820	0	0	0	0	0
ChemDiv-C260-2692	1	1	0	0	0
PubChem-135869534	0	0	0	0	0
PubChem-91487881	0	0	0	0	0
ZINC21525754	0	0	0	0	0
PubChem-16352732	0	0	0	0	0
PubChem-136659176	0	1	1	1	1
PubChem-135405892	0	0	0	0	0
PubChem-126853168	1	0	0	0	0
ZINC65196494	0	0	0	0	0
PubChem-41119771	1	1	0	0	1
ZINC20113894	0	0	0	0	0
PubChem-71600230	0	0	0	0	1
ZINC13365292	0	0	1	1	0
LN00379431	0	0	0	0	0
PubChem-3750748	1	1	0	0	1
ZINC4034881	0	0	0	0	0
ZINC65196500	0	0	0	0	0
ZINC100771598	0	0	0	0	0
PubChem-25352696	0	0	0	0	0
PubChem-91428044	0	0	0	0	0
ZINC33057775	0	0	0	0	0

### 5. Pharmacokinetic proprieties

To predict the pharmacokinetic properties of absorption, distribution, metabolism and excretion (ADME), pkCSM web server was used to calculate the following parameters: water solubility (log mol/L), Caco-2 cell permeability, human intestinal absorption (HIA), blood-brain barrier (BBB) permeability, central nervous system (CNS) permeability and total clearance.

Water solubility (LogS) indicates the solubility of a compound in water at 25°C. Generally, water-soluble drugs are more readily absorbed than lipid-soluble ones. in vitro Caco-2 cell permeability is a crucial measure of drug absorption, with a compound considered to have high Caco-2 permeability if its value surpasses 8 x 10 ^−6^ cm/s. Within the pkCSM model, high Caco-2 permeability aligns with predicted values exceeding 0.90. The intestine typically serves as the primary site for drug absorption from orally administered solutions. A compound with absorbance below 30% is deemed poorly absorbed. An established gauge of blood-brain barrier (BBB) penetration is the log BB ratio, which reflects drug molecule concentrations in the brain and blood. Compounds with log BB > 0.3 exhibit high BBB permeability, while those with log BB < 1.0 show limited BBB distribution. Furthermore, central nervous system (CNS) permeability is a vital parameter for assessing the blood-brain permeability of a drug candidate, expressed as LogPS. Compounds with LogPS <-3 are considered incapable of penetrating the CNS.

Based on these ADME properties, 18 compounds were selected for further analysis. The results for these compounds are detailed in Table 5, while the other parameters are shown in **Table S4** in the supplementary data.

**Table 5.** Some ADME parameters of the top-ranked compounds.

Code	Water solubility	Caco2 permeability	Intestinal absorption (human)	BBB permeability	CNS permeability	Total Clearance
ZINC17286185	-5.117	0.818	83.062	-0.367	-2.784	0.727
ZINC35562518	-4.994	0.819	82.23	-0.389	-2.876	0.732
ZINC64438506	-5.467	1.165	96.008	-0.435	-2.363	0.804
ZINC64438485	-5.241	1.163	97.137	-0.98	-2.573	0.505
PubChem-121231416	-4.14	0.979	94.665	-0.031	-2.006	0.608
ZINC21797190	-4.273	1.083	96.539	-0.669	-2.597	0.623
PUBChem 157251136	-4.222	1.106	94.303	-0.893	-2.705	1.167
PubChem-42810820	-5.266	1.294	94.667	-0.394	-1.924	0.316
ChemDiv-C260-2692	-5.941	1.284	97.247	-0.422	-2.392	0.819
PubChem-91487881	-2.946	1.374	90.351	-0.966	-2.226	0.369
PubChem-126853168	-4.351	1.428	96.515	-0.399	-2.234	0.514
ZINC65196494	-4.278	1.131	98.641	-0.725	-2.5	0.947
PubChem-41119771	-4.601	1.251	95.633	0.709	-1.937	0.039
PubChem-3750748	-5.732	1.102	91.195	0.215	-1.931	-0.313
ZINC4034881	-4.991	1.18	97.628	-0.693	-2.477	0.972
PubChem-25352696	-2.773	1.444	98.767	-0.866	-2.82	0.534
PubChem-91428044	-5.217	1.3	94.768	0.399	-2.503	0.758
ZINC33057775	-4.528	1.202	93.212	-0.615	-2.241	-0.075

### 6. Docking Validation

Docking validation was performed using AutoDock 4.2. The results are presented in Table 6.

**Table 6.** Binding energy (B.E), Intermolecular Energy (I.M.E), Internal Energy (I.E), Torsional Energy (T.E) and constant of inhibition (K.I).

Code	B.E (Kcal/mol)	I.M.E (Kcal/mol)	I.E (Kcal/mol)	T.E (Kcal/mol)	KI (nM)
ChemDiv-C260-2692	-12.43	-14.82	-2.39	2.39	0.768
PubChem3750748	-12.3	-14.69	-2.05	2.39	0.958
PubChem25352696	-10.58	-12.63	-1.06	1.79	17.63
PubChem41119771	-11.23	-13.02	-1.22	1.79	5.91
PubChem42810820	-11.6	-12.79	-0.77	1.19	3.13
PubChem91428044	-10.2	-12.88	-1.1	2.68	33.36
PubChem91487881	-10.71	-13.09	-1.13	2.39	14.15
PubChem121231416	-11.63	-14.32	-0.86	2.68	2.98
PubChem126853168	-12.26	-13.75	-0.93	1.49	1.04
PUBChem157251136	-11.84	-14.23	-1.61	2.39	2.09
ZINC4034881	-11.51	-13.89	-2.1	2.39	3.67
ZINC17286185	-11.41	-14.39	-1.84	2.98	4.36
ZINC21797190	-12.37	-14.45	-1.22	2.09	0.862
ZINC33057775	-12.25	-14.04	-1.64	1.79	1.06
ZINC35562518	-12.22	-14.9	-1.74	2.68	1.11
ZINC64438485	-13.07	-15.76	-1.37	2.68	0.262
ZINC64438506	-13.11	-15.8	-1.37	2.68	0.244
ZINC65196494	-11.7	-13.49	-1.28	1.79	2.65

To identify the top CB1 agonist several criteria were taken into account including energy values (e.g. binding energy or Ki values), interactions with key amino acids in the CB1 binding site, number of hydrogen bonds and distances between hydrogen bonds. These interactions are essential for designing effective and selective CB1 agonists. ^10^ For instance, π-π interactions with aromatic residues (Phe296, Phe170, Phe174, Trp 279) and hydrogen bonds with Ser383 or Thr197 stabilize the agonist-receptor complex, while residues such as Phe379 and Asp366 play a key role in receptor activation through electrostatic interactions. ^10^ Based on these criteria three compounds were identified as CB1 receptor agonists: ZINC64438506, ZINC64438485 and PUBChem157251136.

**Figure 4.**
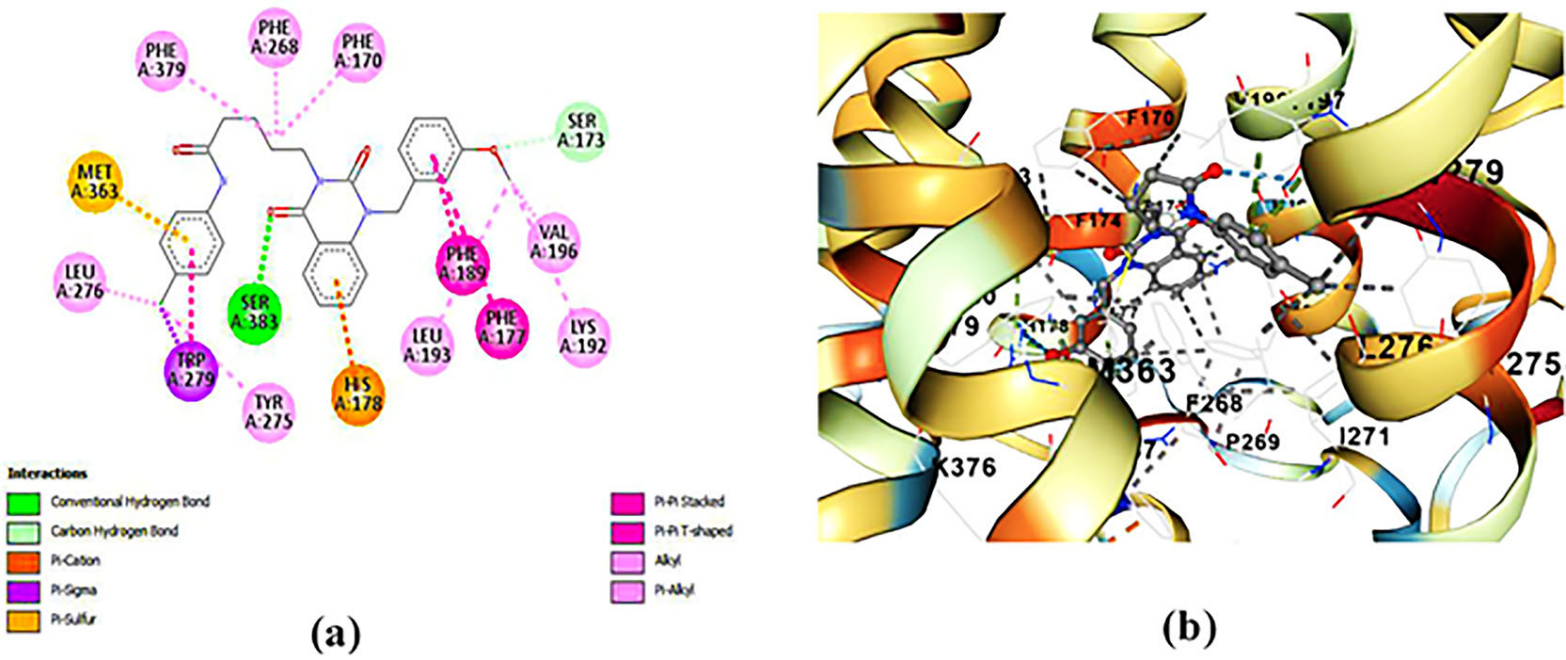
Docking analysis of ZINC64438506 agonist with CB1 receptor. (a) 2D view of binding site interactions (b) 3D view of binding conformation.

**ZINC64438506:** The docking analysis of CB1 receptor and ZINC64438506 selected agonist is shown in Table 7 and Figure 4. The ZINC64438506 agonist was fixed in the CB1 binding pocket (cavity size = 2963 Å) through various type of interactions, including hydrogen bonds with key amino-acid residues SER A: 173 and SER A: 383, hydrophobic interactions with residues PHE A: 108, PHE A: 177, PHE A: 189, LEU A: 193, THR A: 197, PHE A: 268, TYR A: 275, LEU A: 276, TRP A: 279 and PHE A: 379 and π-π interaction with PHE A: 268. These interactions likely contribute to the compound’s low binding affinity (-13.11 Kcal/mol) and low inhibition constant (Ki = 0.244 nM) ( Table 6). The strong binding affinity may be attributed to the presence of short hydrogen bonds with SER A: 173 (3.34 Å) and SER A: 383 (51.79 Å).

**Table 7.** Ligand-receptor interactions of the three highest-ranked agonists.

Hydrophobic interactions			Hydrogen Bonds				π-Stacking			Halogen Bonds		
Residue	AA	Distance	Residue	AA	Distance H-A	Distance D-A	Residue	AA	Distance (Å)	Residue	AA	Distance
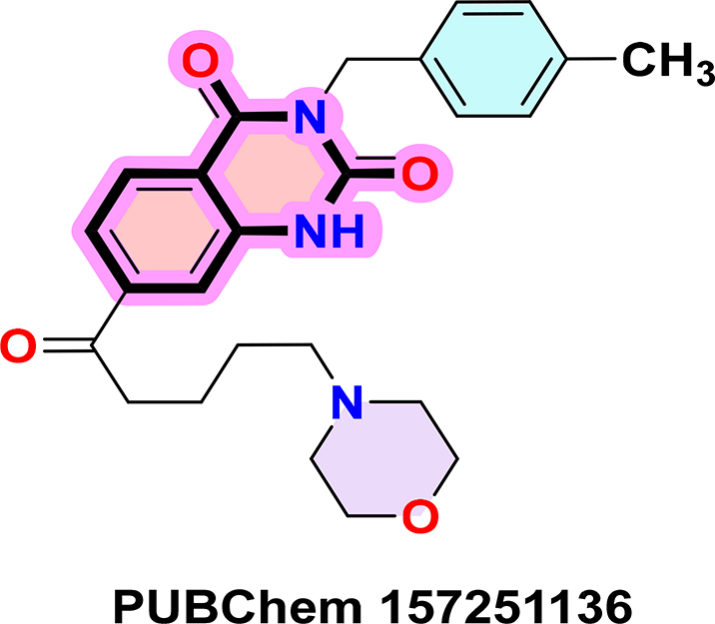												
174A	PHE	3.83	173A	SER	3.23	3.72	268A	PHE	4.71			
174A	PHE	3.70	178A	HIS	1.94	2.93	279A	TRP	3.94			
177A	PHE	3.26	383A	SER	2.04	2.81						
193A	LEU	3.37										
197A	THR	3.13										
268A	PHE	3.48										
276A	LEU	3.98										
279A	TRP	3.60										
379A	PHE	3.49										
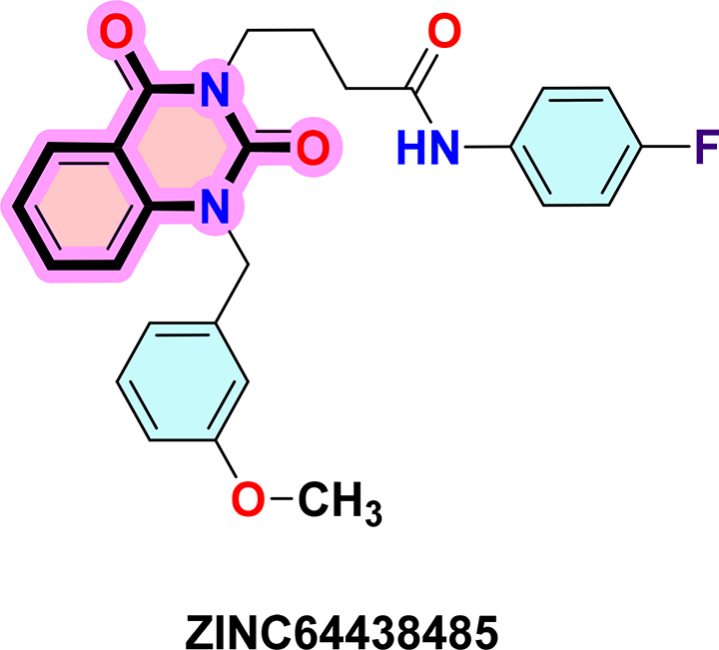												
174A	PHE	3.87	197A	THR	1.94	2.93	174A	PHE	5.09			
177A	PHE	3.68	383A	SER	3.08	3.74	178A	HIS	4.62			
177A	PHE	3.37					268A	PHE	4.77			
177A	PHE	3.88					279A	TRP	4.93			
189A	PHE	3.58										
193A	LEU	3.42										
193A	LEU	3.81										
196A	VAL	3.19										
271A	ILE	3.91										
276A	LEU	3.40										
279A	TRP	3.65										
279A	TRP	3.77										
379A	PHE	3.95										
379A	PHE	3.40										
380A	ALA	3.34										
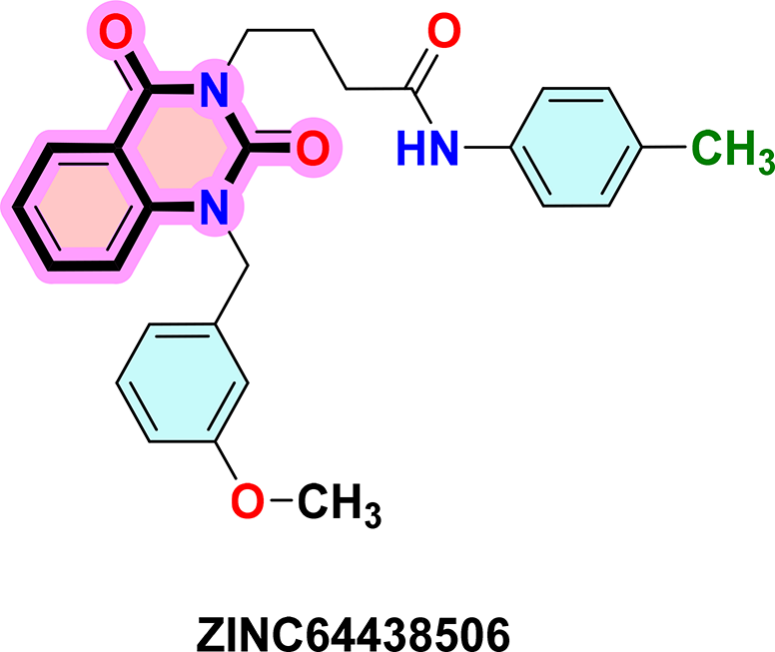												
108A	PHE	3.77	173A	SER	3.34	3.82	268A	PHE	4.77			
177A	PHE	3.82	383A	SER	1.79	2.67						
177A	PHE	3.22										
177A	PHE	3.47										
189A	PHE	3.22										
193A	LEU	3.47										
197A	THR	3.59										
268A	PHE	3.63										
275A	TYR	3.13										
276A	LEU	3.26										
279A	TRP	3.40										
279A	TRP	3.71										
279A	TRP	3.25										
279A	TRP	3.56										
379A	PHE	3.76										

**Figure 5.**
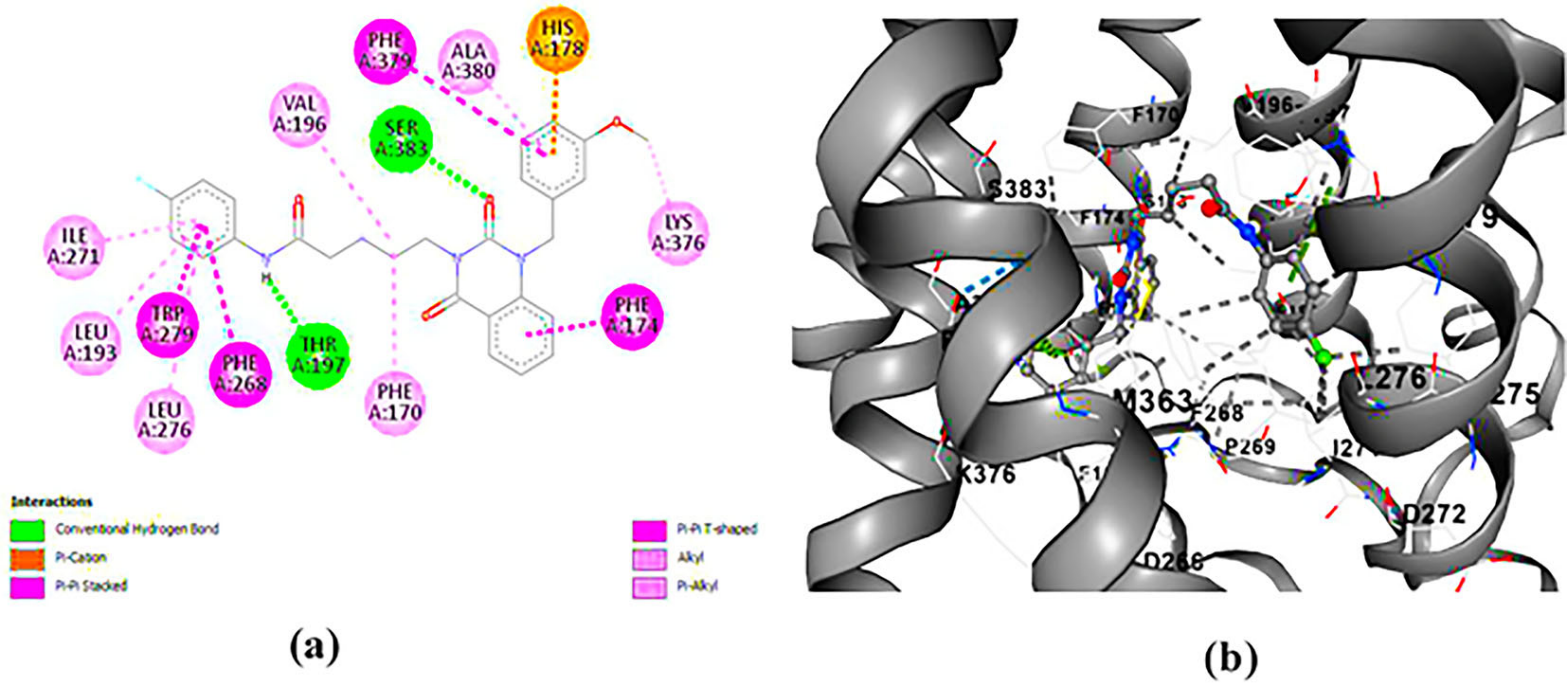
Docking analysis of ZINC64438485 agonist with CB1 receptor. (a) 2D view of binding site interactions (b) 3D view of binding conformation.

**ZINC64438485:** The docking analysis of CB1 receptor and ZINC64438485 selected agonist is shown in Table 7 and Figure 5. The ZINC64438485 agonist was fixed in the CB1 binding pocket (cavity size = 2963 Å) through various type of interactions, including hydrogen bonds with key amino-acid residues THR A: 197 and SER A: 383, hydrophobic interactions with residues PHE A: 174, PHE A: 177, PHE A: 189, LEU A: 193, VAL A: 196 and ILE A: 271 and π-π interaction with PHE A: 174, HIS A: 178, PHE A: 268 and TRP A: 279. These interactions likely contribute to the compound’s low binding affinity (-13.07 Kcal/mol) and low inhibition constant (Ki = 0.262 nM) ( Table 6). The strong binding affinity may be attributed to the presence of short hydrogen bonds with THR A: 197 (1.94 Å) and SER A: 383 (3.08 Å).

**Figure 6.**
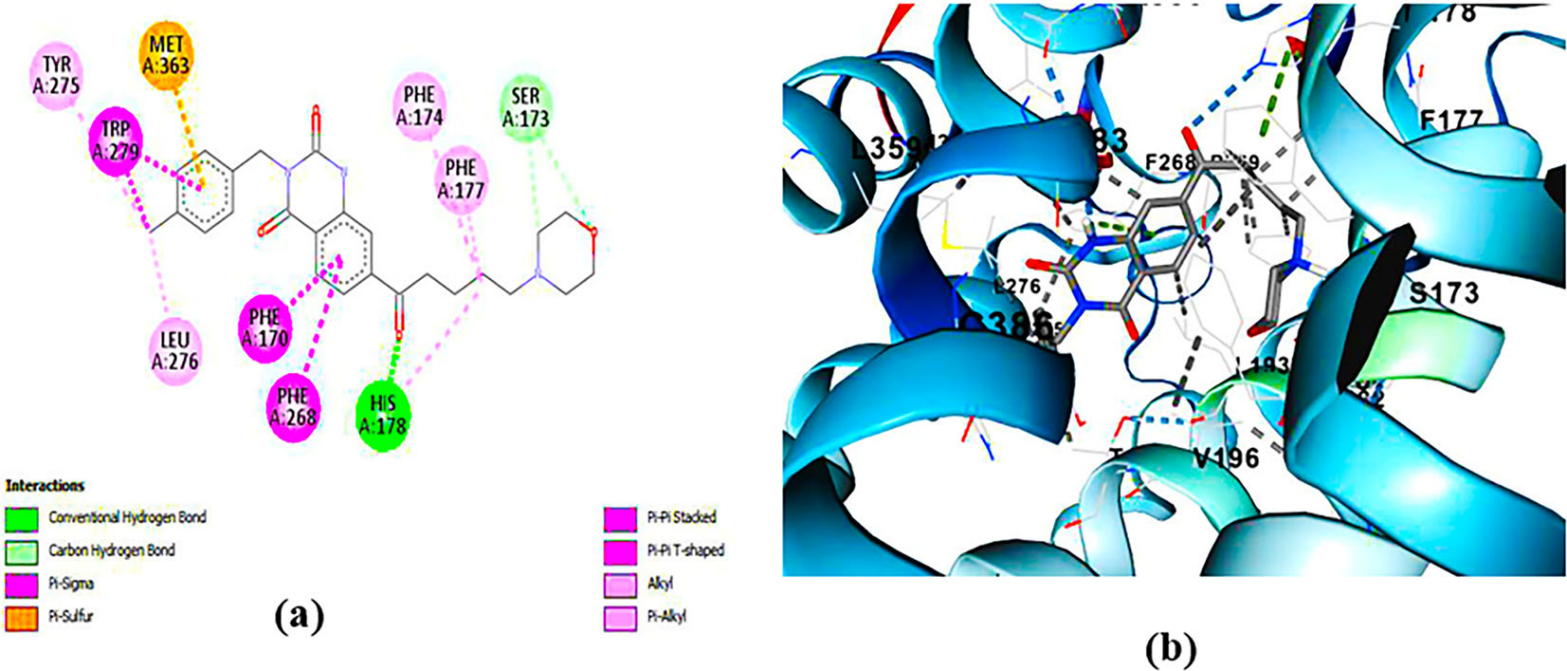
Docking analysis of PUBChem_157251136 agonist with CB1 receptor. (a) 2D view of binding site interactions (b) 3D view of binding conformation.

**PUBChem-157251136:** The docking analysis of CB1 receptor and PUBChem-157251136 selected agonist is shown in Table 7 and Figure 6. The PUBChem157251136 agonist was fixed in the CB1 binding pocket (cavity size = 2963 Å) through various type of interactions, including hydrogen bonds with key amino-acid residues SER A: 173, HIS A: 178 and SER A: 383, hydrophobic interactions with residues PHE A: 174, PHE A: 177, LEU A: 193, THR A: 197, PHE A: 268, LEU A: 276, TRP A: 279 and PHE A: 379 and π-π interaction with PHE A: 268 and TRP A: 279. These molecular interactions likely contribute to the compound’s low binding affinity (-11.84 Kcal/mol) and low inhibition constant (Ki = 2.09 nM) ( Table 6). The strong binding affinity may be attributed to the presence of three hydrogen bonds with SER A: 173 (3.23 Å), HIS A: 178 (1.94 Å) and SER A: 383 (2.04Å).

### 7. MD simulations

**Figure 7.**
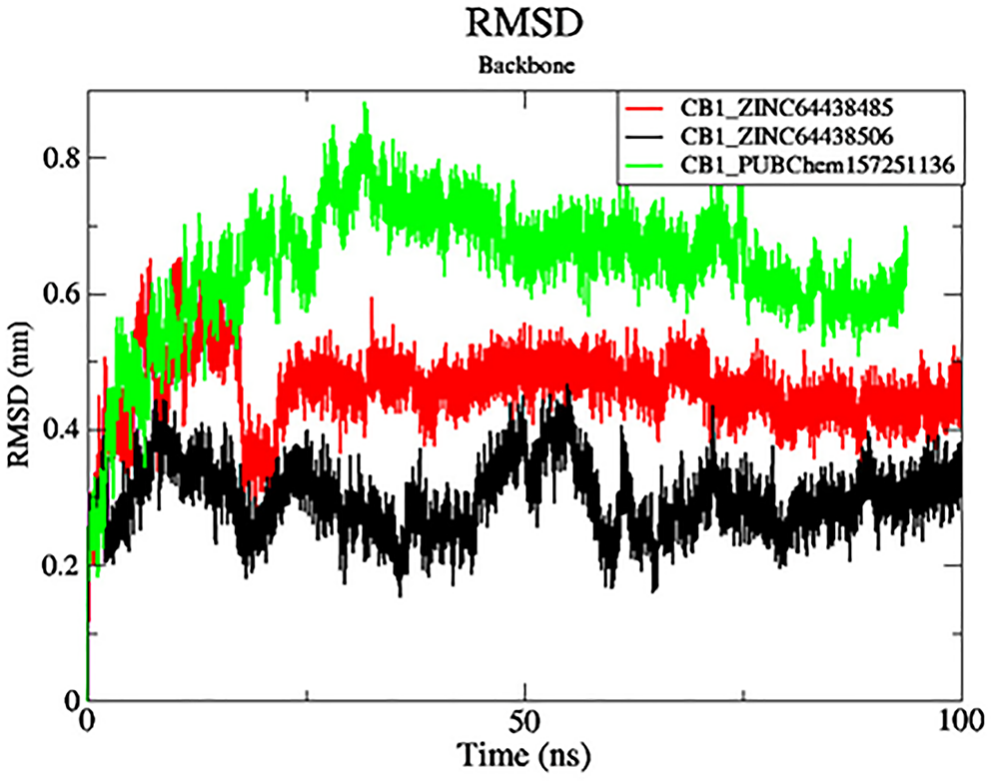
Plots of RMSD over the 100 ns MD simulation. The black color was the CB1_ZINC64438506 complex, the red was the CB1_ZINC64438485 complex and the green was the CB1_PUBChem157251136 complex.

**Figure 8.**
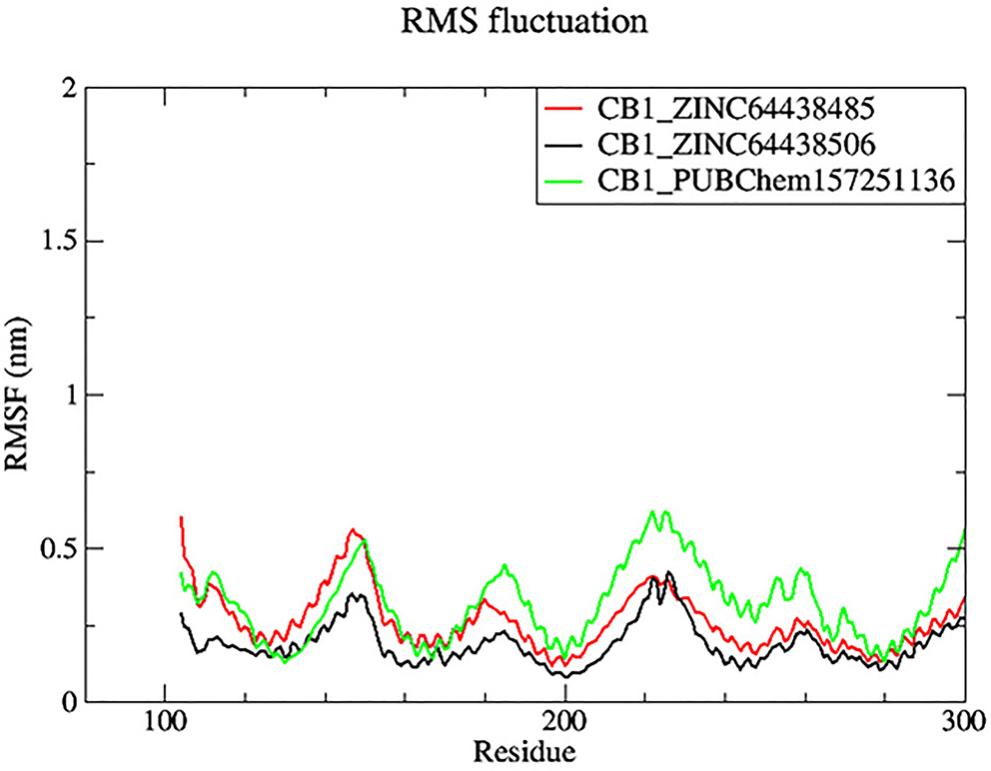
Plots of RMSF over the 100 ns MD simulation. The black color was the CB1_ZINC64438506 complex, the red was the CB1_ZINC64438485 complex and the green was the CB1_PUBChem157251136 complex.

**Figure 9.**
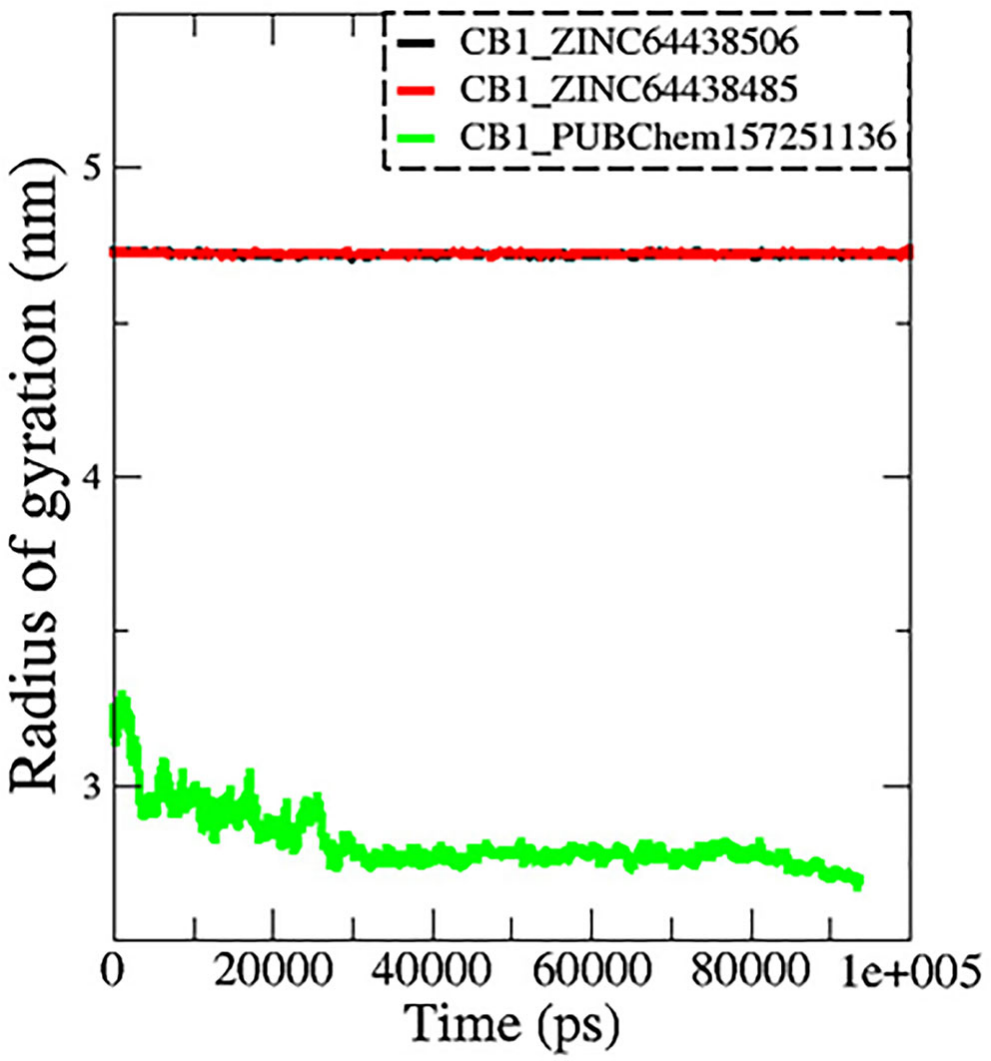
Plots of Rg during the 100 ns of MD simulation. The black color represents the CB1_ZINC64438506 complex, the red color represents the CB1_ZINC64438485 complex and the green color represents the CB1_PUBChem157251136 complex.

**Figure 10.**
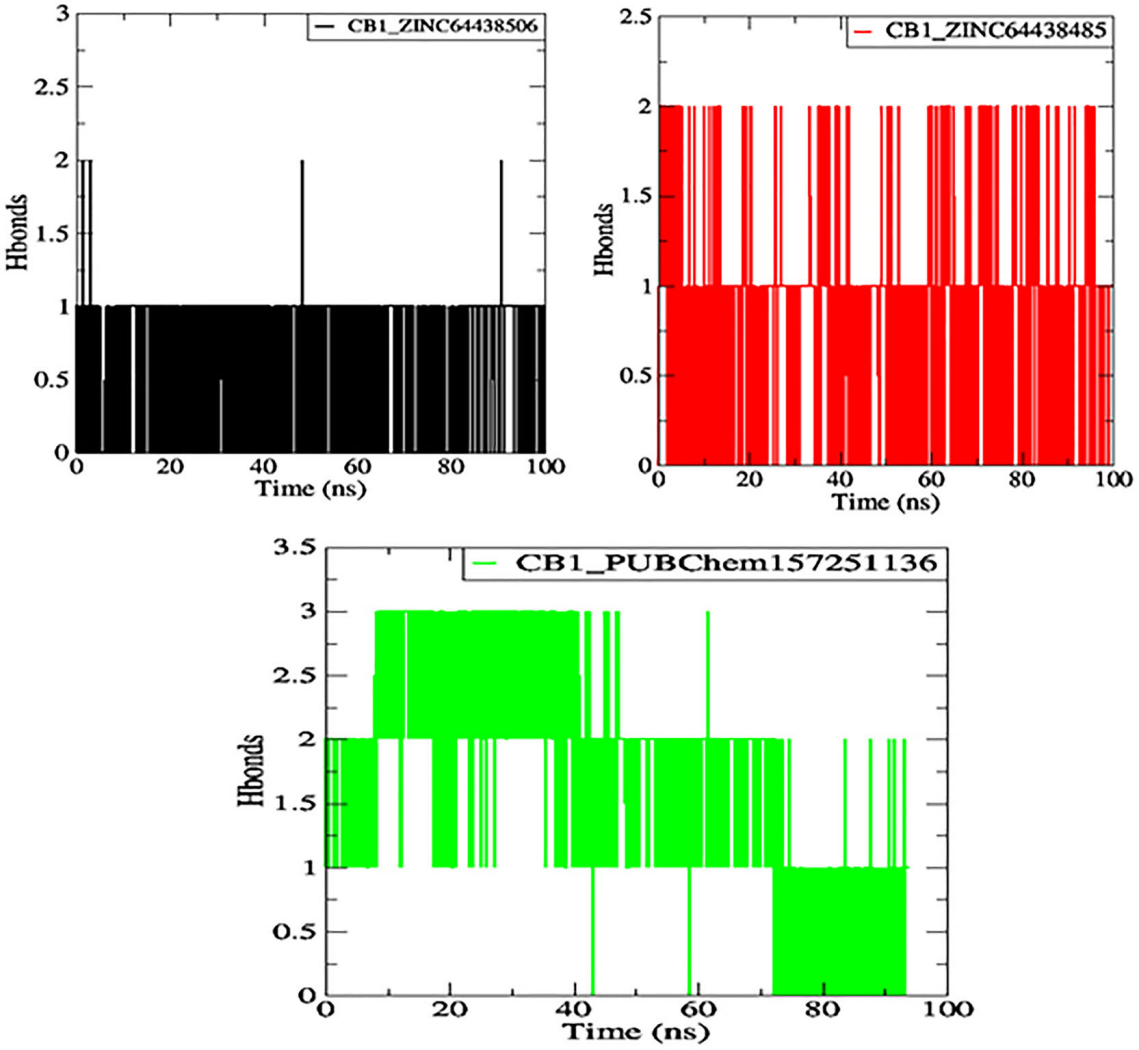
Plot of H-bonds during the 100 ns of MD simulation. The black color represents the CB1_ZINC64438506 complex, the red color represents the CB1_ZINC64438485 complex and the green color represents the CB1_PUBChem157251136 complex.

While molecular docking analyses are enough to understand possible bindings based on ligand positioning and receptor-ligand interactions, it should be noted that such methodologies evaluate the flexibility of the ligand only while keeping the protein in a rigid form. Thus, in order to evaluate the best-docked candidates for binding pose stability and dynamics of protein conformation, molecular dynamics (MD) simulations were performed over 100 ns. The results of the MD simulations, including root-mean-square deviation (RMSD), root-mean-square fluctuation (RMSF), and Ligand-Receptor Interaction Plot analyses, can be found in Figures 7– 10. These studies provide insights into the dynamic behavior and stability of the protein–ligand complexes over time.

**7.1 Root Mean Square Deviation (RMSD)**

RMSD analysis was carried out for the protein backbone to have an idea of each protein-ligand complex’s structural stability during the simulation. ^32, 33^ The results are shown in Figure 7. As shown in Figure 7, mean RMSD values for the CB1_ZINC64438506, CB1_ZINC64438485 and CB1_PUBChem157251136 complex are 0.29 nm, 0.45 nm and 0.64 nm, respectively. The global RMSD values for CB1_ZINC64438506, CB1_ZINC64438485 are small which indicates that these ligands remain stable during the simulation of 100 ns and remained in the binding pocket of the CB1 receptor. The stability of the ligands can be attributed to several strong hydrogen bonds being mediated between these agonists and some of the key amino acids positioned within the binding pocket of the CB1 receptor.

**7.2 Root mean square fluctuation (RMSF)**

Root mean square fluctuation (RMSF) was used to determine the rigid and flexible regions of the CB1 receptor over the 100 ns of MD simulations. ^14^ RMSF has been term definition, instead of just RMSD values, so that the maximum range of motion of a bound ligand can be seen. RMSF is defined as a standard measure of deviation of a molecule from its initial position. ^34^ Molecules and residues should not present a high value, which indicates a flexibility, and those that appears low value has a greater rigidity. The RMSF plot for all complexes ( Figure 8) indicates that most residues located in the CB1 receptor has a low RMSF value, indicating they were rigid and retained stability over the entire 100 ns MD simulation.

**7.3 Radius of gyration (Rg)**

The radius of gyration (Rg) is another important metric measured during MD simulations to determine spatial characteristics of a protein-ligand complex. The radius of gyration is the root mean square distance of all atoms making up a given structure from a relative center common point (the center of mass) and signifies extension versus folding of the given structure. Therefore, a lower Rg would imply stability and a more tightly folded structure, while a higher Rg would imply extension and flexibility of possible structures. In this work, the Rg profiles remained stable over the course of 100 ns of MD simulation for the complexes CB1_ZINC64438506 (mean = 4.70 nm; Figure 9) and CB1_ZINC64438485 (mean = 4.72 nm), while the complex CB1_PUBChem157251136 decreased in Rg (mean = 2.81 nm).

**7.4 Hydrogen bonds**

One of the main factors influencing the affinity of a molecule for the protein binding pocket is its ability to form and maintain hydrogen bonds with the binding site residues. The stability of the selected agonist was assessed by analyzing the hydrogen bonds between the ligand and the protein. The results are presented in Figure 10.

Compounds ZINC64438506, ZINC64438485, and PUBChem157251136 form two, two and three hydrogen bonds with the CB1 binding site, respectively, indicating strong and specific interactions with the protein. These results are consistent with the docking results.

### 8. MM-GBSA calculation for the top three ranked compounds

The binding free energy of all complexes was calculated to revalidate the binding affinity obtained from molecular docking analysis. The results are presented in Table 8. A ΔG _bind_ value below -7 Kcal/mol indicates strong binding, a value between -5 and -7 kcal/mol suggests moderate binding, and a value between -2 and -5 kcal/mol corresponds to weak binding. ^35^ The results show that all the agonists exhibit exceptionally low binding free energies, indicating a remarkable affinity for the target protein.

**Table 8.** MM-GBSA calculations for the top three ranked compounds.

Complex	ΔG _GAS_ (Kcal/mol)	ΔG _SOLV_ (Kcal/mol)	ΔG _bind_ (Kcal/mol)
CB1_ZINC64438506	-80.76	30.99	-49.77
CB1_PUBChem157251136	-107.10	76.51	-30.59
CB1_ZINC64438485	-82.29	32.31	-49.98

**Figure 11.**
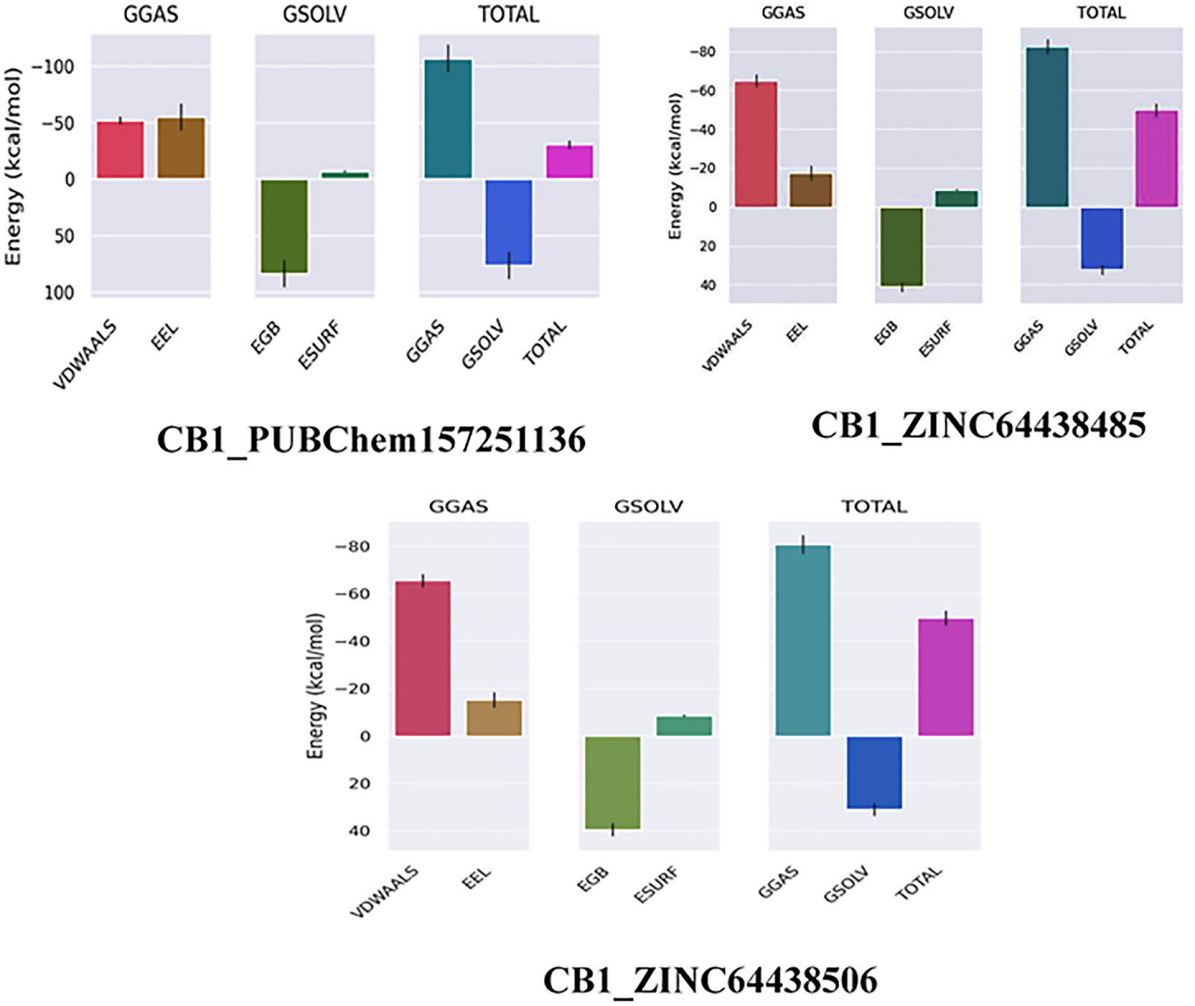
Binding free energy plot of the three highest ranked agonists.

The results also highlight the crucial role of Van der Waals and electrostatic interactions in mediating protein-ligand binding throughout the molecular dynamics simulations ( Figure 11).

### 9. Quinazoline-2,4(1H,3H)-dione derivatives

**Figure 12.**
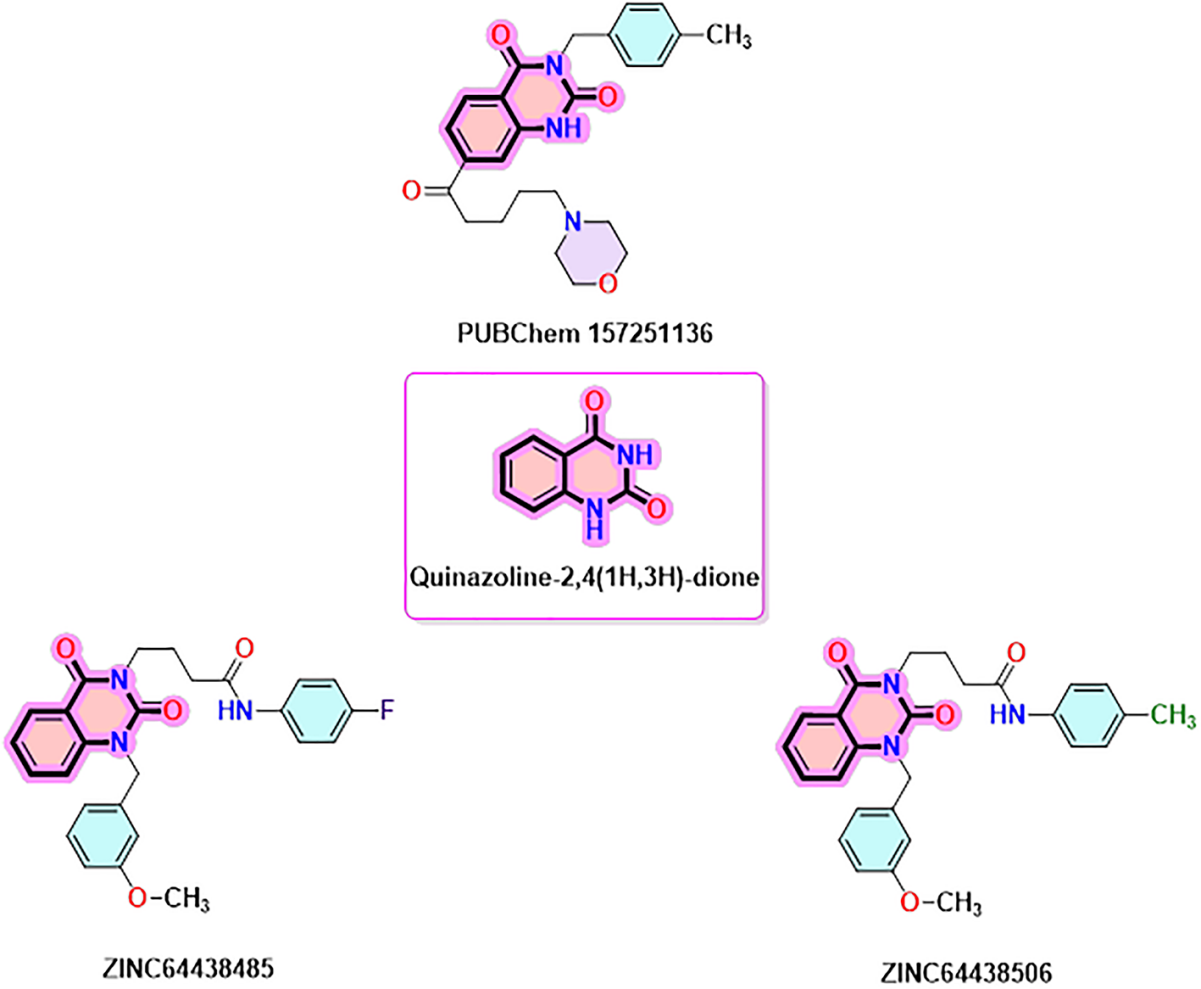
Quinazoline-1, 4(1H, 3H)-dione scaffold and the three highest-ranked agonists (ZINC64438506, PUBChem157251136 and ZINC64438485).

Quinazoline-2,4(1H,3H)-dione derivatives represent a highly promising class of heterocyclic compounds with broad therapeutic potential, particularly as anticancer, ^36– 38^ antibacterial, ^39, 40^ antihypertensive, ^41^ phosphodiesterase (PDE) 4 inhibition, ^42^ 5-HT3A receptor antagonist, ^43^ anti-inflammatory, ^44^ and anan up-and-comingtiviral agents. ^45^ Their fused benzopyrimidinedione scaffold provides an excellent pharmacophore for targeting key biological pathways ( Figure 12). According to the article’s findings, quinazoline-2,4(1H,3H)-dione derivatives may be the first of a new class of CB1 agonists.

### 10. Different synthesis routes of substituted quinazoline-2,4-dione scaffold

**Figure 13.**
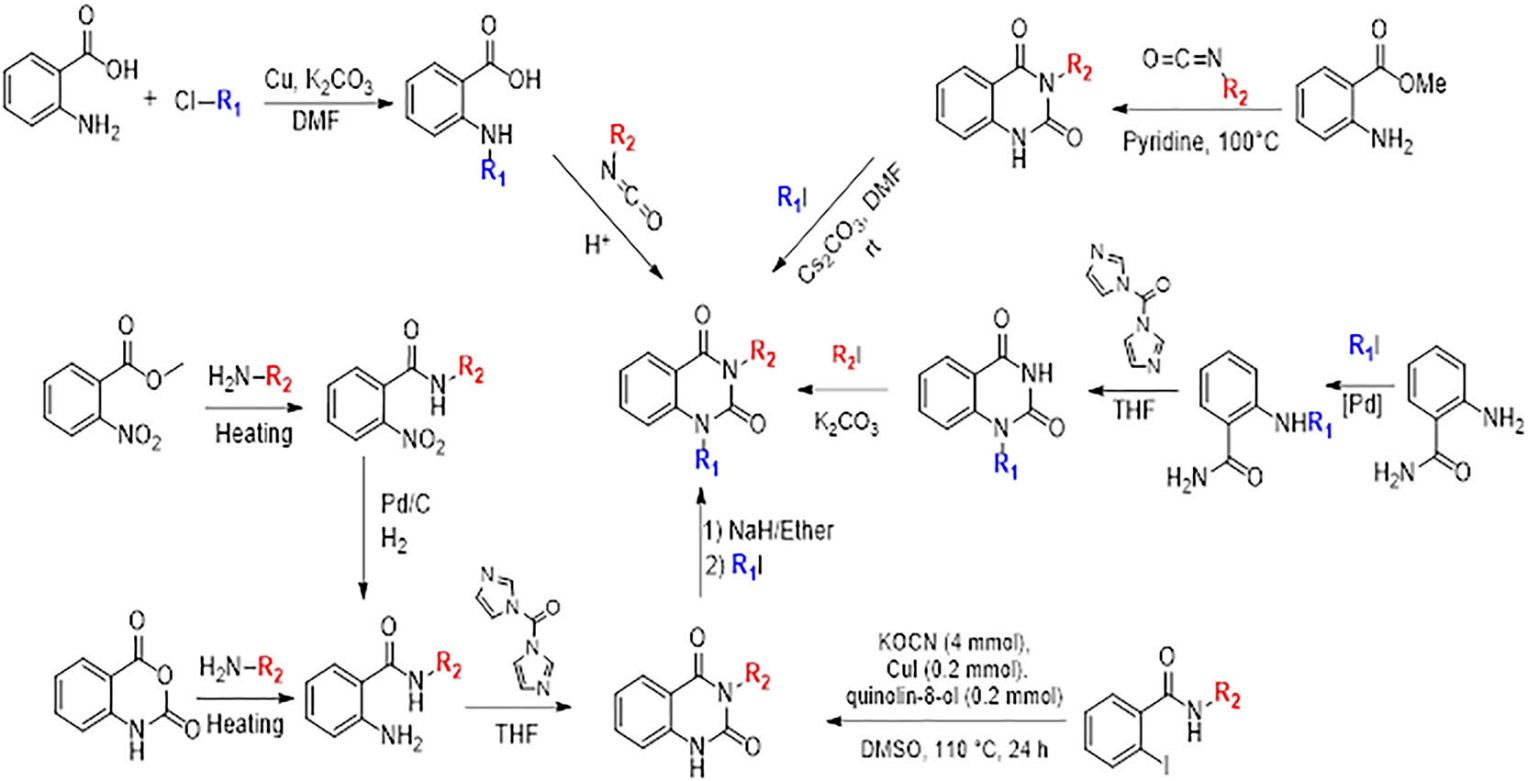
Different synthesis routes of substituted quinazoline-2, 4-dione.

To synthesise the substituted quinazoline-2,4-dione and the highest-ranking agonists identified by virtual screening, namely ZINC64438506, PUBChem157251136 and ZINC64438485, several plausible synthesis strategies were developed based on established methodologies. ^43^ These routes use various precursors such as 2-aminobenzoic acid, ^44^ methyl 2-nitrobenzoate, 2-iodobenzamides ^46^ or 2H-benzo [d][1,3]oxazine-2,4(1H)-dione ^47^ ( Figure 13). The choice of starting material is guided by both its availability and synthetic feasibility. Depending on the substrate, the desired compounds can be obtained by catalytic transformations, particularly transition metal-catalysed couplings, or by intramolecular cyclisation reactions that form the characteristic quinazoline skeleton. The synthetic flexibility offered by these precursors allows the reaction conditions to be adjusted to optimise yield and purity, making them suitable candidates for further pharmacological evaluation and development.

To synthesise substituted quinazoline-2,4-dione and the top-ranked agonists identified by virtual screening - namely ZINC64438506, PUBChem157251136 and ZINC64438485 - several plausible synthetic strategies have been devised, based on established methodologies. ^46^ These routes use various precursors such as 2-aminobenzoic acid, ^47^ methyl 2-nitrobenzoate ^48^ which undergoes catalytic hydrogenation under mild and green conditions (1 atm of H _2_ in ethanol at room temperature), ^49, 50^ 2-iodobenzamides ^51^ or 2H-benzo [d][1,3]oxazine-2,4(1H)-dione ^52^ ( Figure 13). The choice of starting material is guided by both availability and synthetic feasibility. Depending on the substrate, the desired compounds can be accessed by catalytic transformations, including transition metal-catalysed couplings, or by intramolecular cyclisation reactions that form the characteristic quinazoline scaffold. The synthetic flexibility offered by these precursors allows reaction conditions to be fine-tuned to optimize yield and purity, making them suitable candidates for further pharmacological evaluation and development.

## IV. Conclusion

In this study, pharmacophore-based virtual screening was conducted to identify the best CB1 agonist based on the pharmacophoric features of AM11542. The optimal pharmacophore model comprised two hydrogen bond acceptors (HBA), one aromatic ring (AR), and two hydrophobic centers (HY). This model was subsequently applied to screen a database of more than five million compounds, including Molprot with 4,742,020 molecules, ChEMBL34 with 2,264,112 molecules, ZINC with 13,127,550 molecules, ChemDiv with 1,456,120 molecules, ChemSpace with 50,181,678 molecules, Enamine with 4,117,328 molecules, MCULE with 39,843,637 molecule, MCULE-ULTIMATE with 126,471,502 molecules, NCI Open Chemical Repository with 52,237 molecules, LabNetwork with 1,794,286 molecules and PubChem with 103,302,052 molecules. Based on the generated pharmacophore model, 433 compounds were selected for further evaluation. Subsequent molecular docking refined this selection to 61 high-affinity ligands (≤-9.00 kcal/mol), which were refined to 18 promising leads through ADME-Tox analysis. Among these, three compounds were selected as potential agonists of the CB1 receptor based on their binding affinity and strong interactions with key binding pocket residues (Ser383, Ser173, His178, and Thr197). Molecular dynamics simulations using Gromacs 2024.4 confirmed the structural stability of these complexes, with low RMSD (<1 nm) and RMSF (<1 nm) values, indicating minimal conformational fluctuations. MM-GBSA calculations further validated the thermodynamic stability, with binding free energies ranging from -30.59 to -49.98 kcal/mol, reinforcing their potential as potent CB1 agonists. The three selected compounds shared a common quinazolinz-2,4(1H,3H)-dione scarfed, indicating that derivatives of this structure could pave the way for developing new CB1 receptor agonists.

## Data Availability

No underlying data are associated with this article. Repository name: “Quinazoline-2,4(1H,3H)-dione derivatives as new class of CB1 Agonists: A ppharmacophore-based virtual screening workflow and Lead discovery” https://doi.org/ 10.5281/zenodo.17274156. ^53^ This project contains the following extended data:
•
**Supplementary Table 1**. (Binding free energies of the 433 top-ranked compounds from pharmacophore-based virtual screening) •
**Supplementary Table 2.** (Computationally predicted toxicity profiles of top 61 compounds) •
**Supplementary Table 3.** (Physicochemical properties of studied compounds) •
**Supplementary Table 4.** (Some ADME parameters of the top-ranked compounds) Data are available under the terms of the Creative Commons Attribution 4.0 International license (CC-BY 4.0).
