# Supplementary material for: Quinazoline-2,4(1H,3H)-dione derivatives as new class of CB1 Agonists: A pharmacophore-based virtual screening workflow and drug discovery
Source: F1000Res. 2026 Mar 11;14:1322. Originally published 2025 Nov 27. [Version 3] doi: 10.12688/f1000research.171433.3 (PMC12754364; doi:10.12688/f1000research.171433.3)
Supplement: Peer review history

# Reviewer report: Reviewer response for version 3

**DOI:** 10.5256/f1000research.197019.r466648

**Published:** 2026-03-19

**Authors:** Samir Chtita, Bouchra Rossafi

**Affiliations:** 1 Institution: Hassan II University of Casablanca, Morocco, Morocco, Morocco

The authors have addressed all of my recommendations, and I recommend the indexing of the article

Is the work clearly and accurately presented and does it cite the current literature?

Yes

If applicable, is the statistical analysis and its interpretation appropriate?

Not applicable

Are all the source data underlying the results available to ensure full reproducibility?

Yes

Is the study design appropriate and is the work technically sound?

Yes

Are the conclusions drawn adequately supported by the results?

Yes

Are sufficient details of methods and analysis provided to allow replication by others?

Partly

Reviewer Expertise:

Molecular modeling

We confirm that we have read this submission and believe that we have an appropriate level of expertise to confirm that it is of an acceptable scientific standard.

# Reviewer report: Reviewer response for version 2

**DOI:** 10.5256/f1000research.194643.r449401

**Published:** 2026-02-03

**Authors:** Samir Chtita, Bouchra Rossafi

**Affiliations:** 1 Hassan II University of Casablanca, Morocco, Morocco

The manuscript titled **"** ***Quinazoline-2,4(1H,3H)-dione derivatives as new class of CB1 Agonists: A pharmacophore-based virtual screening workflow and drug discovery*** **",** present an in silico investigation aimed at identifying new CB1 agonists derived from quinazoline-2,4(1H,3H)-dione scaffolds, employing a pharmacophore-based virtual screening strategy followed by molecular docking, MD simulations and MMGBSA calculations. While the study provides interesting results, some aspects require improvement, including:

1. The authors should more clearly demonstrate the novelty and originality of the present study in the Introduction section, particularly in comparison with previously reported CB1 agonists and related computational studies.
2. After the pharmacophore-based virtual screening, the authors proceeded with molecular docking. However, the rationale behind the selection of 61 compounds with a docking score threshold of −9 kcal/mol as top molecules is not clearly justified. The authors should explain why this specific cutoff was chosen.
3. Although a reference agonist (AM11542) was used for the generation of the pharmacophore model, it is not clear whether this compound was subsequently used as a reference drug in the docking, ADMET and MD analyses.
4. In the context of the targeted pathology and the central role of the CB1 receptor, the analysis of BBB permeability and central nervous system CNS parameters is highly relevant. These aspects should be more thoroughly analyzed and discussed.
5. The Introduction identifies several key residues involved in CB1 receptor activation. However, it is not clearly demonstrated whether the identified compounds effectively interact with these key residues.
6. Comparing MM-GBSA results with docking scores or reference compounds could strengthen the interpretation.
7. The quality and clarity of the docking figures should be improved.
8. **In the table 2, the unit for binding energy should be written as “kcal” instead of “Kcal” to follow standard scientific notation. **
9. The manuscript would benefit from a dedicated section discussing the limitations of the study.
10. Experimental validation of the identified compounds perspectives should be included in the Conclusion.
11. Information included on the different modeling techniques in material and methods section is quite trivial and should be more detailed. To enhance the introduction of the computational section, it is recommended to provide a detailed discussion and comparison of different methods as reported in the literature. In order to facilitate a better understanding for readers, the following previous studies employing docking and molecular dynamic in this research area can be cited: Refer to reference 1, 2, 3, & 4

Is the work clearly and accurately presented and does it cite the current literature?

Yes

If applicable, is the statistical analysis and its interpretation appropriate?

Not applicable

Are all the source data underlying the results available to ensure full reproducibility?

Yes

Is the study design appropriate and is the work technically sound?

Yes

Are the conclusions drawn adequately supported by the results?

Yes

Are sufficient details of methods and analysis provided to allow replication by others?

Partly

Reviewer Expertise:

Molecular modeling

We confirm that we have read this submission and believe that we have an appropriate level of expertise to confirm that it is of an acceptable scientific standard, however we have significant reservations, as outlined above.

## References

[1] : Rational design of novel pyridine-based drugs candidates for lymphoma therapy. *Journal of Molecular Structure* .2022;1270: 10.1016/j.molstruc.2022.133964 10.1016/j.molstruc.2022.133964

[2] : In vitro and in silico evaluation of the antimicrobial and antioxidant activities of spiropyrazoline oxindole congeners. *Arabian Journal of Chemistry* .2024;17(1) : 10.1016/j.arabjc.2023.105465 10.1016/j.arabjc.2023.105465

[3] : An In Silico Study Based on QSAR and Molecular Docking and Molecular Dynamics Simulation for the Discovery of Novel Potent Inhibitor against AChE. *Pharmaceuticals* .2024;17(7) : 10.3390/ph17070830 10.3390/ph17070830 PMC1128038139065681

[4] : Rational identification of small molecules derived from 9,10-dihydrophenanthrene as potential inhibitors of 3CLpro enzyme for COVID-19 therapy: a computer-aided drug design approach. *Structural Chemistry* .2022;33(5) : 10.1007/s11224-022-02004-z 1667-1690 10.1007/s11224-022-02004-z PMC926118135818588

# Response

**Published:** 2026-02-15

**Authors:** ABDELLAH EL AISSOUQ

**Affiliations:** Sidi mohamed ben abdellah university, Morocco

reponse to Q1: your remarks have been taken into account in the revised manuscript

 response to Q2: this threshold was chosen based on the reference molecule (see sup data)

 response to Q3: the agonist (AM11542) was selected as the reference coumpound for the whole study

 response to Q4-9: your remarks have been taken into account in the revised manuscript

 response to Q10: all selected compounds are known drugs

# Reviewer report: Reviewer response for version 1

**DOI:** 10.5256/f1000research.189041.r439371

**Published:** 2025-12-30

**Authors:** Mourad Fawzi

**Affiliations:** 1 Faculty of Sciences Semlalia, Cadi Ayyad University, Department of Chemistry, Laboratory of Molecular Chemistry, Marrakech, Morocco

The study entitled " ***Quinazoline-2,4(1H,3H)-dione derivatives as new class of CB1 Agonists: A pharmacophore-based virtual screening workflow and Lead discovery*** " presents a pharmacophore-based virtual screening of a large database aimed at developing new CB1 agonists. I believe this work is suitable for publication in ***F1000 Research*** before the minor revisions below:

**Comments for the authors:**

1. Page 17: "form two, two and three hydrogen bonds with the CB1 binding site, respectively, …" – The word "two" is repeated; please remove one.
2. Some abbreviations are not clearly defined in the manuscript, such as **GPCR **and **ICL3**. Please ensure all abbreviations are spelled out upon first use.
3. Page 19: The sentence ˝Translated with DeepL.com (free version)˝  should be removed.
4. More details about the advantage of work in conclusion are required
5. Grammatical errors and typos are present throughout the manuscript. A thorough proofreading is recommended to improve clarity and language quality.
6. Regarding **Table 6**: The reported **Kᵢ** values indicate that the screened compounds exhibit promising drug-like properties. Therefore, I suggest modifying the title to better reflect this finding. Proposed revision:

" ***Quinazoline-2,4(1H,3H)-dione derivatives as new class of CB1 Agonists: A pharmacophore-based virtual screening workflow and Lead discovery*** "

Is the work clearly and accurately presented and does it cite the current literature?

Yes

If applicable, is the statistical analysis and its interpretation appropriate?

I cannot comment. A qualified statistician is required.

Are all the source data underlying the results available to ensure full reproducibility?

No source data required

Is the study design appropriate and is the work technically sound?

Yes

Are the conclusions drawn adequately supported by the results?

Partly

Are sufficient details of methods and analysis provided to allow replication by others?

Yes

Reviewer Expertise:

Bioinformatics, Organic Chemistry, Medicinal Chemistry

I confirm that I have read this submission and believe that I have an appropriate level of expertise to confirm that it is of an acceptable scientific standard.

# Response

**Published:** 2026-01-01

**Authors:** ABDELLAH EL AISSOUQ

**Affiliations:** Sidi mohamed ben abdellah university, Morocco

**Reviewer #2:**

 The study entitled "Quinazoline-2,4(1H,3H)-dione derivatives as new class of CB1 Agonists: A pharmacophore-based virtual screening workflow and drug discovery" presents a pharmacophore-based virtual screening of a large database aimed at developing new CB1 agonists. I believe this work is suitable for publication in F1000 Research before the minor revisions below:

 Comments for the authors:

1. Page 17: "form two, two and three hydrogen bonds with the CB1 binding site, respectively, …" – The word "two" is repeated; please remove one.
2. Some abbreviations are not clearly defined in the manuscript, such as GPCR and ICL3. Please ensure all abbreviations are spelled out upon first use.
3. Page 19: The sentence ˝Translated with DeepL.com (free version)˝ should be removed.
4. More details about the advantage of work in conclusion are required
5. Grammatical errors and typos are present throughout the manuscript. A thorough proofreading is recommended to improve clarity and language quality.
6. Regarding Table 6: The reported Kᵢ values indicate that the screened compounds exhibit promising drug-like properties. Therefore, I suggest modifying the title to better reflect this finding. Proposed revision:

**Response to Questions 1-6:** We thank the reviewer for their insightful comments. All remarks have been addressed in the revised manuscript."

# Reviewer report: Reviewer response for version 1

**DOI:** 10.5256/f1000research.189041.r437306

**Published:** 2025-12-29

**Authors:** Fatima En-nahli

**Affiliations:** 1 University of Moulay Ismail, Meknes, Morocco

Dear Editor,

 I would like to thank you for the opportunity to review the manuscript entitled *“Quinazoline-2,4(1H,3H)-dione derivatives as a new class of CB1 Agonists: A pharmacophore-based virtual screening workflow and Lead discovery.”* The study addresses a relevant topic and provides valuable insights into CB1 agonist identification through an in silico workflow. Overall, the manuscript is promising; however, I believe that **minor revisions** are required to improve clarity and scientific rigor.

 Please find below my main remarks for the authors:

1. The current title would benefit from being reformulated to make it more concise and more attractive to readers. A clearer title highlighting the novelty and the integrated workflow is recommended.
2. The authors mention the use of molecular dynamics (MD) simulations in the Materials and Methods section, but MD is not mentioned in the Abstract. For coherence and completeness, this important component of the workflow should be briefly stated in the Abstract.
3. In the graphical abstract, the authors indicate two docking steps (first using AutoDock Vina, then AutoDock4). It would be helpful to briefly clarify the rationale behind this double-docking strategy and how each tool contributes to the screening workflow.
4. The authors are encouraged to integrate a comparative discussion between the pharmacophore hits, docking scores, and the stability of key amino acids involved in ligand recognition, supported by MD fluctuation analysis (e.g., RMSF). This would reinforce the consistency and predictive power of the multi-step in silico approach.
5. Since none of the proposed candidates were synthesized or experimentally validated, the authors should clearly state the value of the in silico-only approach and discuss its limitations. It is also recommended to suggest potential collaboration with experimental researchers for future synthesis and biological evaluation.
6. Given that the identified compounds appear to be promising drug candidates, it would be appropriate to add a short section in the conclusion outlining perspectives for future work.

Overall, the manuscript represents an interesting contribution, and after addressing these minor points, it will be suitable for publication.

 Thank you again for entrusting me with the review of this manuscript.

 Kind regards,

Is the work clearly and accurately presented and does it cite the current literature?

Yes

If applicable, is the statistical analysis and its interpretation appropriate?

Yes

Are all the source data underlying the results available to ensure full reproducibility?

Yes

Is the study design appropriate and is the work technically sound?

Yes

Are the conclusions drawn adequately supported by the results?

Yes

Are sufficient details of methods and analysis provided to allow replication by others?

Yes

Reviewer Expertise:

chimie informatique

I confirm that I have read this submission and believe that I have an appropriate level of expertise to confirm that it is of an acceptable scientific standard.

# Response

**Published:** 2026-01-01

**Authors:** ABDELLAH EL AISSOUQ

**Affiliations:** Sidi mohamed ben abdellah university, Morocco

**Reviewer #1:**

 Dear Editor, I would like to thank you for the opportunity to review the manuscript entitled *“Quinazoline-2,4(1H,3H)-dione derivatives as a new class of CB1 Agonists: A pharmacophore-based virtual screening workflow and Lead discovery.”* The study addresses a relevant topic and provides valuable insights into CB1 agonist identification through an in silico workflow. Overall, the manuscript is promising; however, I believe that **minor revisions** are required to improve clarity and scientific rigor. Please find below my main remarks for the authors:

 1. The current title would benefit from being reformulated to make it more concise and more attractive to readers. A clearer title highlighting the novelty and the integrated workflow is recommended.

**Response to Q1:** the title has been changed to “Quinazoline-2,4(1H,3H)-dione derivatives as new class of CB1 Agonists: A pharmacophore-based virtual screening workflow and drug discovery”

 2. The authors mention the use of molecular dynamics (MD) simulations in the Materials and Methods section, but MD is not mentioned in the Abstract. For coherence and completeness, this important component of the workflow should be briefly stated in the Abstract.

**Response to Q2: **The molecular dynamics (MD) simulations are mentioned in the abstract, as shown in the following excerpt from the abstract section “Molecular dynamics simulations (100 ns, GROMACS) demonstrated structural stability (RMSD < 1 nm) and low conformational flexibility (RMSF < 1 nm) for all complexes. MM-GBSA binding free energy calculations further confirmed the thermodynamic stability of all complexes, with interaction energies ranging from -30.59 to -49.98 kcal/mol.

 3. In the graphical abstract, the authors indicate two docking steps (first using AutoDock Vina, then AutoD ock4).It would be helpful to briefly clarify the rationale behind this double-docking strategy and how each tool contributes to the screening workflow.

**Response to Q3:** We thank the reviewer for raising this important point regarding our docking strategy. The double-docking approach was implemented to enhance the robustness and reliability of our virtual screening workflow.

 4. The authors are encouraged to integrate a comparative discussion between the pharmacophore hits, docking scores, and the stability of key amino acids involved in ligand recognition, supported by MD fluctuation analysis (e.g., RMSF). This would reinforce the consistency and predictive power of the multi-step in silico approach.

 5. Since none of the proposed candidates were synthesized or experimentally validated, the authors should clearly state the value of the in silico-only approach and discuss its limitations. It is also recommended to suggest potential collaboration with experimental researchers for future synthesis and biological evaluation.

 6. Given that the identified compounds appear to be promising drug candidates, it would be appropriate to add a short section in the conclusion outlining perspectives for future work.

**Response to Q4, 4 and 6** : The identified compounds are promising drug candidates
